# Metabolic fingerprint of insulin resistance in human polymorphonuclear leucocytes

**DOI:** 10.1371/journal.pone.0199351

**Published:** 2018-07-13

**Authors:** Martina Palomino-Schätzlein, Rafael Simó, Cristina Hernández, Andreea Ciudin, Pablo Mateos-Gregorio, Antonio Hernández-Mijares, Antonio Pineda-Lucena, José Raúl Herance

**Affiliations:** 1 Structural Biochemistry Laboratory, Centro de Investigación Príncipe Felipe, Valencia, Spain; 2 Diabetes and Metabolism Research Unit, Vall d’Hebron Research Institute, Barcelona, Spain; 3 CIBERDEM (Instituto de Salud Carlos III), Madrid, Spain; 4 Service of Endocrinology, University Hospital Doctor Peset, Foundation for the Promotion of Health and Biomedical Research in the Valencian Region (FISABIO), Valencia, Spain; 5 Drug Discovery Unit, Instituto de Investigación Sanitaria La Fe, Hospital Universitario y Politécnico La Fe, Valencia, Spain; 6 Medical Molecular Imaging Research Group, Vall d’Hebron Research Institute, CIBBIM-Nanomedicine, CIBERbbn, Barcelona, Spain; Medical University of Vienna, AUSTRIA

## Abstract

The present study was aimed at determining the metabolic profile of PMNs in obese subjects, and to explore its potential relationship with insulin resistance (IR). To achieve this goal, a pilot clinical study was performed using PMNs from 17 patients with obesity and IR, and 17 lean controls without IR, which was validated in an additional smaller cohort (consisting of 10 patients and 10 controls). PMNs were isolated from peripheral blood and nuclear magnetic resonance was used to perform the metabolomic analysis. A total of 48 metabolites were quantified. The main metabolic change found in PMNs was a significant increase in 2-aminoisobutyric acid with a direct correlation with HOMA-IR (p<0.001), BMI (p<0.000001) and waist circumference (p<0.000001). By contrast, a decrease of 3-hydroxyisovalerate was observed with an inverse correlation with HOMA-IR (p = 0.001), BMI (p = 0.001) and waist circumference (p = 0.0001). Notably, the metabolic profile in plasma was different than that obtained in PMNs. In summary, our results suggest that the change in 3-hydroxyisovalerate and 2-aminoisobutyric is the key metabolic fingerprint in PMNs of obese subjects with IR. In addition, our methodology could be an easy and reliable tool for monitoring the effect of treatments in the setting of precision medicine.

## Introduction

Obesity represents a major public health problem and is associated with a significant economic burden in the health systems of developed countries, mainly due to associated comorbidities such as type 2 diabetes and cardiovascular disease [1,2]. Adipose tissue is considered an endocrine organ that regulates the innate and adaptive immune response by the production of adipokines such as leptin and cytokines. One of the characteristics of obesity is its association with insulin resistance (IR), systemic low-grade inflammation and a deregulation of the immune system [3,4].

The discovery of new biomarkers able to identify obese patients at risk of developing comorbidities and the gaining of new insights into the complex relationships between the factors contributing to the disease are crucial for the design of personalized medicine programs. In this regard, the study of polymorphonuclear cells (PMNs) could be very useful because they are first immune cells that respond to inflammation by being recruited into adipose tissue [5]. They could play a role in initialising the inflammatory cascade in response to obesity by producing chemokines and cytokines, facilitating macrophage infiltration and inducing IR [6]. There is evidence that insulin regulates glucose metabolism in PMNs and is responsible for the activation of its main functions [7] which may be altered by the IR associated with either obesity or type 2-diabetes [8,9]. It should be noted that euglucemic hyperinsulinemic clamp performed in healthy subjects resulted in a significant increase of PMN functions such as chemotaxis, phagocytosis and bactericidal capacities. Therefore, insulin can modulate PMN functions not just by the attainment of better metabolic control [7]. This finding supports the critical role of IR in neutrophil impairment, thus raising the question of whether intracellular neutrophil metabolic changes could be a useful tool for research purposes.

Omics techniques have become a powerful approach, widely adopted for clinical diagnostics and with a crucial role in unravelling the molecular mechanisms involved in pathology [10]. Unlike genes and proteins, whose functions are subject to epigenetic regulation and post-translational modifications respectively, metabolites serve as direct signatures of biochemical activity and are therefore easier to correlate with the phenotype [11]. Since blood is easily accessible in routine clinical practise, the main focus of metabolomics has been based on the analysis of circulating metabolites from plasma or serum, and several metabolites has been identified as potential biomarkers of obesity [12,13]. However, much less is known regarding metabolites contained within circulating blood cells in terms of increasing our understanding of the pathophysiology of these cells in disease or in biomarker discovery. Neutrophils are the most abundant PMN cells in human blood circulation, playing an essential role in the immunological responses of the body in pathology.

Among all the possible techniques for detecting metabolites, nuclear magnetic resonance (NMR) has proven to be a powerful tool for studying the metabolic alterations associated with pathological conditions [14]. NMR data stand out for their high reproducibility, allowing the generation of very robust models that can integrate data from different analytical platforms and can be applied over longer periods of time [15]. Furthermore, NMR has the advantage of providing complete structural information regarding compounds, so allowing the identification of new or unexpected metabolites, which is crucial for the biochemical interpretation of cellular metabolism. On the other hand, the relatively low sensitivity of NMR spectroscopy, one of its main drawbacks, has been significantly improved in recent years by the introduction of cold probes [16]. Although several studies based on mass spectrometry regarding the metabolic profiling of leucocytes in diseases unrelated to obesity have recently been reported [17,18], to the best of our knowledge NMR spectroscopy, widely applied for blood plasma and serum [19] has not previously been explored for the analysis of leucocytes.

On this basis, the main aim of the present study was to perform the first global examination of the metabolic profile of PMN cells in obese patients by NMR, and to explore its potential relationship with IR.

## Material and methods

### Ethics statement

The study was conducted according to the guidelines laid down in the Declaration of Helsinki, and the Ethics Committees of the University Dr. Peset Hospital (protocol number CEIC140/14) and Vall d’Hebron University Hospital (protocol number PR(AG) 86/2013) approved all procedures. Written informed consent was obtained from all the participants.

### Study subjects

A case-control study was designed which included 17 morbid obese individuals (body mass index [BMI] ≥37.50 Kg/m^2^) recruited at the Outpatient’s Department of the Endocrinology Service of Dr. Peset University Hospital in Valencia. The control group consisted of 17 age-matched non-smoking subjects with a BMI≤ 25 Kg/m^2^.

The study was conducted according to the guidelines laid down in the Declaration of Helsinki, and the Ethics Committee of the Dr. Peset University Hospital (protocol number CEIC140/14). Written informed consent was obtained from all the participants. Smokers and patients with diabetes or previous cardiovascular events or other comorbidities were excluded from the study. The reason to exclude diabetic patients was to study the relationship between IR and the PMN metabolic profile without the background noise of hyperglycemia and all the metabolic pathways related to diabetes. In order to validate the study, a smaller cohort of obese (n = 10) and non-obese control subjects (n = 10) recruited in the Unit of Obesity at Vall d’Hebron University Hospital were included. Written consent was obtained from all the participants and the study was approved by the Ethics Committee of the Vall d’Hebron University Hospital (protocol number PR(AG) 86/2013). Details about validation patients can be found in the S5 Table.

Blood samples were collected under fasting conditions, stored at 4°C and processed within the first 2 hours. Several anthropometrical parameters and biochemical analyses including glucose, high-density lipoproteins (HDL), low-density lipoproteins (LDL), triglycerides (TG), the BMI, waist, insulin, and the homeostasis model assessment of insulin resistance (HOMA-IR) were measured.

### Isolation of PMN from peripheral blood

Ten mL of peripheral blood freshly extracted from obese and normal individuals were poured into a quartz tube with 20 mL of Ficoll and left standing for 20 minutes until 2 phases separated by gravity. The top ring consisted of a mixture of leucocytes and the bottom ring of erythrocytes. The leukocyte ring was transferred in a tube with the same volume of Ficoll, care being taken not to mix them, and centrifuged at 300 g and 20° C for 25 min to obtain a pellet of PMN cells. To minimize the contamination of the PMN pellet with the remaining erythrocytes, it was treated with 1 ml of erythrocyte lysis buffer for 5 minutes and centrifuged at 300 g and 20 °C for 5 min. Then, the resulting pellet was resuspended in the same volume of PBS, centrifuged at 2000 g and 20 °C for 5 min, and the supernatant was discarded. This washing operation was repeated. Cell counting was performed using Tuerk staining solution and the purity was tested with CD15 by cytometry. For storage, 160 μL of ice-cold methanol were added per 10 million cells and the samples frozen directly at -80 °C.

### Extraction of polar metabolites

Metabolites were extracted following the methanol-chloroform-water protocol that was recently optimized for PMN cells [20, 21]. Briefly, frozen samples were placed on ice and allowed to thaw for 5 min. 80 μL of chloroform at 4°C were added (solvent volumes are indicated for 10 million cells). After 10 min, the samples were homogenized with a vortex, resuspended with a pipette and transferred to a plastic tube. For uniform cell breakage, the samples were placed in liquid nitrogen for 1 min and then allowed to thaw on ice for 2 min. This step was repeated twice. Afterwards, 125 μL of distilled water and 125 μL of chloroform were added and the sample vortexed. Then, the samples were centrifuged at 13000 g for 20 min at 4°C to separate the phases. The upper phase water/methanol was separated from the interphase and lyophilized for 2 hours to remove water and methanol. Sample extracts were stored at -80 °C until the preparation of the samples for NMR experiments. Before NMR analysis, frozen samples were placed on ice and allowed to thaw for 5 min. 550 μl of phosphate buffer (100 mM Na_2_HPO_4_ pH 7.4, in D_2_O) containing 0.1 mM deuterated trimethylsilylpropanoic acid (internal standard) were added and samples were transferred to a 5 mm NMR tube. The samples were stored at 4 °C until 15 min before analysis and analysed the same day.

### Preparation of plasma samples for metabolomics analysis

Five mL of peripheral blood freshly extracted were transferred to plasma tubes and allowed to stand for 30 min. The supernatant was then collected and stored at -80°C until NMR measurement. At the time of analysis, the plasma samples were thawed on ice. 300 μl of 10% D_2_O buffer (5 mM TSP, 140 mM Na_2_HPO_4_, 0.04% NaN_3_, pH 7.4) were added to 300 μl of plasma sample. After this, 550 μl of the mixture were transferred to a 5-mm NMR tube for analysis.

### Solvents and reagents

Unless otherwise indicated, the solvents and reagents employed were purchased from Sigma-Aldrich (Madrid, Spain), Scharlab (Sentmenat, Spain), Falcon BD (Madrid, Spain), Labclinics (Barcelona, Spain), or Eurisotop (Gif sur Yvette, France) and were used in the form in which they were supplied. Gases were supplied by Air-Liquide (Valencia, Spain).

### Nuclear magnetic resonance (NMR) experiments

NMR spectra were recorded at 27 °C on a Bruker AVI-600 using a 5 mm TCI cryoprobe and processed using Topspin3.2 software (Bruker Biospin). For PMN extracts, ^1^H 1D NMR spectra with water presaturation (25 Hz) and a noesy mixing time of 10 ms were acquired with 256 free induction decays (FIDs). 64k data points were digitalized over a spectral width of 30 ppm for an optimal baseline correction. A 4s relaxation delay was incorporated between FIDs. The FID was multiplied by an exponential function with a 0.5 Hz line broadening factor. For plasma samples, a Carr–Purcell–Meiboom–Gill (CPMG) spin-echo pulse sequence, which generates spectra edited by T2 relaxation times with reduced signals from high molecular weight species and improved resolution of low molecular weight metabolite resonances, was acquired with a total of 16 accumulations and 72 K data points over a spectral width of 16 ppm. A 4-second relaxation delay was included between FIDs and a water presaturation pulse of 25 Hz was applied.

The parameters for 2D experiments were 512 increments in t1 and 32 FIDS for total correlation spectroscopy (TOCSY) experiments with MLEV pulse sequence, and 256 t1 increments and 96 FIDS for HSQC (Heteronuclear Single Quantum Correlation) experiments. Both experiments had a relaxation delay of 1.5 s and were acquired in the phase-sensitive mode. The mixing time for TOCSY spectra was set to 65 ms.

### Data analysis

Signals in the ^1^HNMR spectra were assigned to the corresponding metabolites with the help of 2D Experiments, spectral databases HMBD (Human Metabolome Database) [22] and the Biological Magnetic Resonance Bank (BMRB) [23]. In ambiguous cases, the assignment was confirmed by spiking the spectra with reference compounds. Spectra were normalized to total intensity to minimize the differences in concentration and experimental error during the extraction process. Optimal integration regions were defined for each metabolite, an attempt being made to select the signals without overlapping (S1 Table). Integration was performed with Global Spectra Deconvolution (GSD) in MestreNova 8.1.

For multivariate analyses, metabolite tables generated from spectra integration were univariate scaled (each value being divided by the standard deviation of each variable) and mean centred for an easier interpretation of the data and to take the variation of small signals into account. Principal component analysis (PCA) and projection on latent structure-discriminant analysis (PLS-DA) analysis was performed with SIMCA-P 13.0 (Umetrics, Sweden). PLS-DA models were validated by permutation and cross validation analysis. Random Forest and receivers operating characteristic (ROC) curves were performed with Metaboanalyst web server 3.0. ROC curves were performed with MonteCarlo cross-validation using balanced subsampling and Random Forest was selected as the classification method.

For the correlation of metabolite concentrations with biochemical and anthropometrical parameters, a Spearman’s rank correlation coefficient analysis was performed using R commander (The R Foundation). Spearman’s rank correlation coefficients (rho) > 0.6 or < -0.6 and p ≥ 0.001 were considered significant between variables.

For the biological interpretation of the results and the identification of metabolic pathways the Kegg Data Base and MetPA (Metaboanalyst) were used.

Normality tests, t-tests and beeschwarm plots were performed with unscaled, normalized concentration data in R (The R Foundation).

## Results

### Subjects

The main biochemical and anthropometrical clinical features of morbid obese patients and age-matched controls are shown in Table 1 and S2 Table. As expected, the BMI, waist circumference and HOMA-IR were significantly higher in the obese group than in normal controls (Table 1). In addition, HDL-cholesterol was lower in the obese group and the atherogenic index was higher in the obese individuals.

**Table 1:** Comparison of anthropometrical and biochemical parameters of obese patients and healthy individuals.

	Healthy individuals (n = 17)	Obese individuals (n = 17)	P*
Age	41±2	41±3	0.9862
BMI, kg/m2	22.37±0.61	45.5±1.42	8.57e-10
Waist, cm	86±2	129±3	1.02e-10
Glucose, mg/dL	95±4	96±3	0.5578
HDL, mg/dL	45±2	37±1	0.0272
LDL, mg/dL	113±9	112±8	0.5640
TG, mg/dL	106±11	138±13	0.2125
Atherogenic index	0.05±0.1	0.06±0.1	0.0173
Insulin, mg/dL	8.1±0.7	18.8±1.8	1.20e-05
HOMA	2.01±0.23	4.45±0.44	2.83e-05

BMI: body max index; HDL: high-density lipoproteins; LDL: low-density lipoproteins; TG: triglycerides; atherogenic index = logTG/HDL; HOMA: homeostatis model assessment. Data are mean±SEM.

*P values were calculated with two tailed Mann-Whitney U-test. Alpha levels were set to 0.05.

To detect potential outliers, principal component analysis (PCA) was performed of all individuals using as input variables age, BMI, waist, HDL, LDL, TG, insulin, HOMA-IR and glucose. The PCA score and loading plot as well the Distance to Model X plot, revealed that no outliers were present (S1–S3 Figs).

### Metabolomic profile of PMN cells

**Fig 1:**
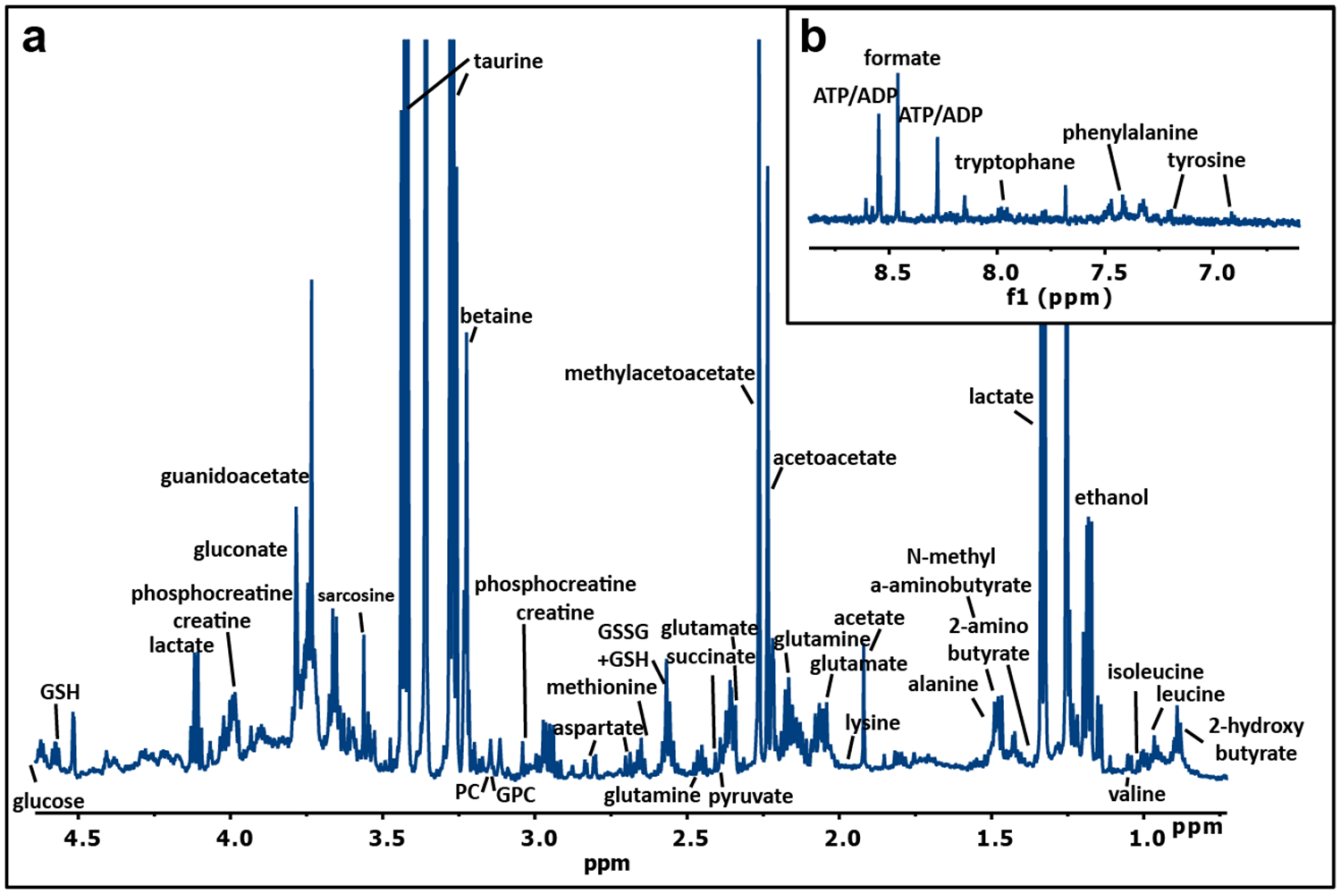
^1^H NMR spectrum of PMN cells. ^1^H NMR spectrum of an aqueous extract of PMN cells from a normal individual acquired at 27 °C with a 600 MHz spectrometer equipped with a cryoprobe. The most significant spectral regions are shown: a) aliphatic region, b) aromatic region.

PMN samples of the two cohorts were analysed following a recent approach described by our group [21], represented in S4 Fig. Briefly, PMNs were first isolated from peripheral blood by a double Ficoll-Paque gradient, with purity higher than 90% tested by flow cytometry being obtained. The differences between samples were minimal and non-statically significant. Metabolites were then extracted and the resulting samples analysed by NMR spectroscopy. A representative spectrum of the aqueous extract obtained from PMN cells is shown in Fig 1, where 48 different polar metabolites could be identified and assigned using NMR databases, 2D-NMR spectra and standards. These metabolites correspond to amino acids, sugars, organic acids and nucleotides, among others (Fig 1, S2 Table).

### Altered metabolic profile of PMN cells in obese patients

**Fig 2:**
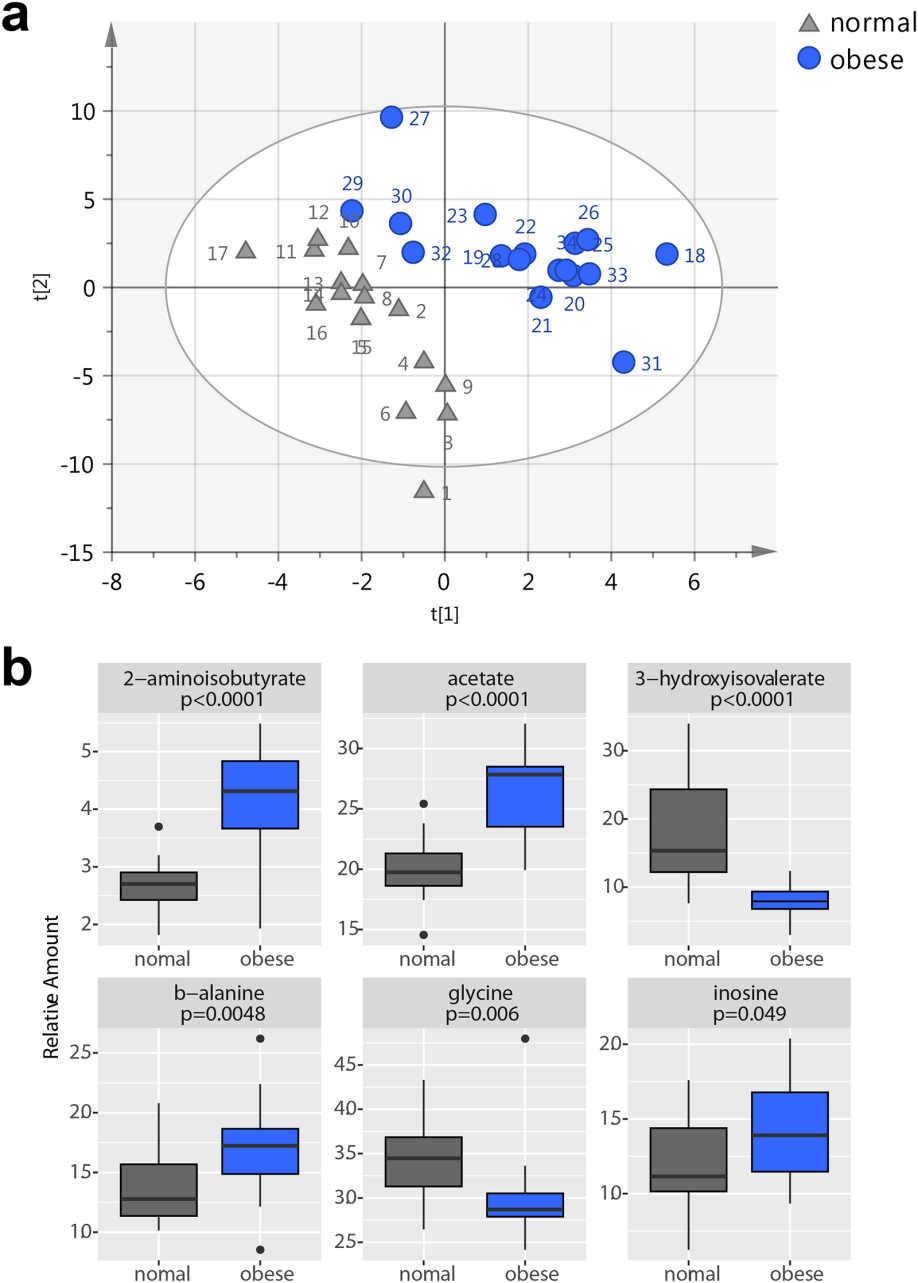
a) Plots from PLS-DA and PLS analysis of the metabolite profile of PMN cells from cohorts. PLS-DA score plot from PLS-DA analysis of the metabolic profile of PMN cells from samples of normal individuals (triangle) and obese patients (circle). R2Y(cum) = 0.812, Q2 (cum) = 0.547. 2 components. p-value from cross validation ANOVA = 0.0005. b) Significant Metabolites. Beeswarm plots for the most significant metabolites found in the comparison between obese and normal individuals. Concentration values are normalized to total area. P values were calculated from the Man Whitney t-test.

To gain new insights into the changes in the metabolic profile of obese subjects, NMR profiles of all PMN samples were analysed using multivariate analysis (Normalized raw data can be found in S3 and S4 Tables). PCA was used as a first approximation to identify clustering trends between samples and outliers. Although no clear grouping between the cohorts was detected in the score plot of the first two principal components, a clear clustering could be observed in the fourth component (S5 Fig), indicating that differences between the groups existed, but were masked by the general variability between samples. Therefore, a supervised discrimination model was built with PLS-DA, with two components and high goodness of fit and prediction values (R2Y(cum) = 0.812, Q2 (cum) = 0.547) (Fig 2A). A permutation test proved the absence of overfitting (S6 Fig), and a good predictive capacity was confirmed by cross validation. This robust separation between cohorts provided evidence that the metabolic profile of neutrophils from obese patients and individuals with normal weight was different. Random Forest classification provided class errors of 0.059 for normal and 0.176 for obese subjects, corresponding to a sensitivity of 82% and a specificity of 94%.

Metabolites that were responsible for discrimination between PMN cells from obese and normal individuals were identified in the corresponding loading plot (S7 Fig) and the features selected by Random Forest analysis. The integration values of these metabolites were subjected to Mann-Whitney non-parametric univariate analysis to confirm their significance and represented as beeswarm-plots (Fig 2B). These analyses revealed that the metabolic profile associated with obese patients was characterized by increased levels of 2-aminoisobutyrate, β-alanine, inosine and acetate, and decreased levels of 3-hydroxyisovalerate and glycine. ROC curves based on random forest models gave AUC values of 0.93 (2-aminoisobutyric acid), 0.92 (acetate), 0.91 (3-hydroxyisovalerate), 0.83 (glycine), 0.78 (β-alanine) and 0.7 (inosine).

To further test the correlation of the identified compounds with different biochemical and anthropometrical parameters such as the BMI and waist circumference, as well as HOMA-IR, non-parametric Spearman’s correlations were performed (Table 2). The results revealed a direct correlation between 2-aminoisobutyric acid and HOMA-IR (rho 0.61, p = 0.0004), BMI (rho = 0.79 p = 0.0000007) and waist circumference (rho = 0.75, p = 000000023). Acetate was also directly correlated with HOMA-IR (rho = 0.49, p = 0.007), BMI (rho = 0.67, p = 0.00003) and waist circumference (rho = 0.63, p = 0.0001). By contrast an inverse correlation was detected between 3-hydroxyisovalerate and HOMA-IR (rho = -061, p = 0.000324), the BMI (rho = -0.59, p = 0.0003) and waist circumference (rho = 0.67, p = 0.00004) (Table 2).

**Table 2:** Spearman’s correlation of PMNs metabolites with altered biochemical and anthropometrical parameters of the cohorts included in the study.

		Waist	HOMA-IR	Insulin	BMI	HDL
3-hydroxyisovalerate	rho	-0.67	-0.61	-0.69	-0.59	0.36
	p	4.34e-5	3.24e-4	2e-5	3.59e-4	4.27e-2
β-alanine	rho	0.43	0.28	0.26	0.50	-0.15
	p	1.49e-2	0.13	0.17	3.08e-3	0.40
inosine	rho	0.27	0.24	0.22	0.33	-0.11
	p	0.14	0.19	0.24	5.42e-02	0.55
glycine	rho	-0.46	-0.22	-0.31	-0.40	0.15
	p	9.70e-3	0.23	8.81e-2	2.04 e-2	0.40
2-aminoisobutyric acid	rho	0.75	0.61	0.61	0.79	-0.37
	p	2.30e-7	4.20e-4	3.10e-4	7.0e-7	4.03e-2
acetate	rho	0.63	0.49	0.52	0.67	-0.24
	p	1.71e-4	7.106e-3	3.47e-3	3.20e-5	0.19

Glycine and β-alanine were related to the BMI and waist circumference but not with HOMA-IR.

**Fig 3:**
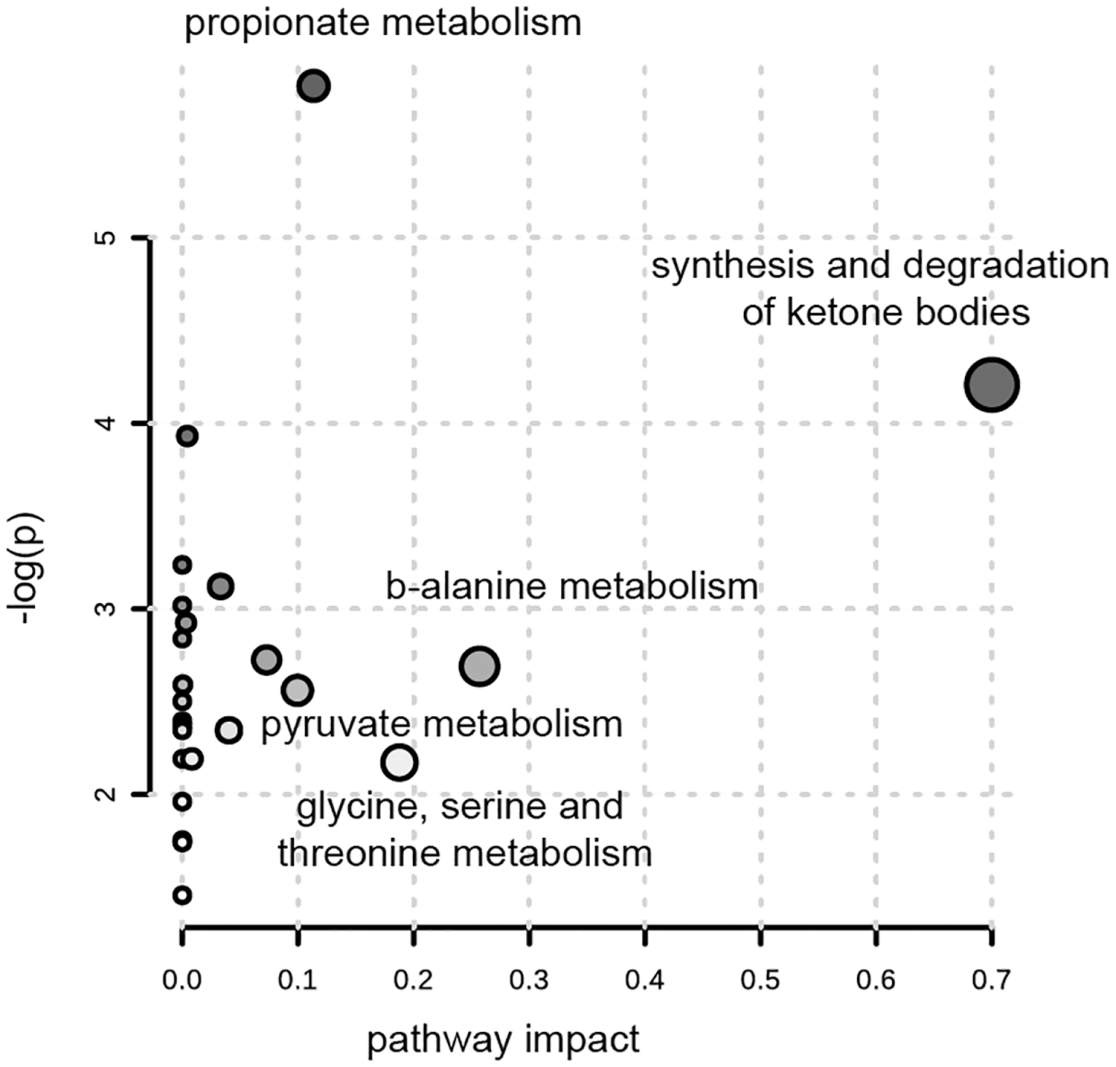
Pathway analysis. Pathway analysis with MetPa showing an overview of the most significantly altered pathways.

An integrative analysis of the results carried out with MetPa, a tool for pathway analysis and visualization, revealed that the most affected metabolic pathways were ketone bodies, β-alanine, glycine, serine, threonine, pyruvate and propionate metabolism (Fig 3).

### Validation of PMN profiling

In order to confirm the metabolic differences detected in the pilot study, we performed an external validation with a small set of samples collected from a new, independent cohort of patients (n = 10). The clinical parameters of these patients can be found in S5 Table. The same analysis and data treatment protocol was performed with these samples, and normalized concentration values of the previously detected significant metabolites (3-hydroxyisovalerate, β-alanine, inosine, glycine, 2-aminoisobutyrate, acetate) were compared. Results are summarized in S6 Table. Differences in 3-hydroxyisovalerate, 2-aminoisobutyrate and glycine could be clearly reproduced, while changes in inosine, β-alanine and acetate were less significant. Thus, even though these results need to be confirmed by studying bigger cohorts of samples, the first three metabolites are the most promising biomarker candidates.

### Alterations in the metabolic profile of plasma of morbid obese patients

As a complementary analysis, the metabolic profile of plasma samples from the same individuals was analysed, following standard NMR metabolomics procedures. This approach permitted us to examine whether our findings were a genuine metabolic fingerprint of obesity and IR detected in PMN or, by contrast, a more generalized systemic effect.

Multivariate analysis confirmed the absence of outliers (S8 Fig). However, no robust separation could be obtained. Univariate analysis identified valine, phospholipids, and N-acetyl groups of plasma proteins as metabolites that were significantly altered in obese individuals (S5 Table). Spearman’s correlations were also performed to relate these metabolites with the clinical parameters as assessed with PMNs. The results are summarized in Table 3. N-acetyl groups of plasma proteins were directly correlated with HOMA-IR, the BMI and waist circumference. By contrast phospholipids were inversely correlated with HOMA-IR, the BMI, waist circumference and HDL-cholesterol.

**Table 3:** Spearman’s correlation of plasma metabolites with altered biochemical and anthropometrical parameters of the cohorts included in the study.

		Waist	HOMA-IR	Insulin	BMI	HDL
Valine	rho	0.311	0.317	0.279	0.284	0.0541
	p	0.108	0.115	0.168	0.136	0.789
N-acetyl groups of plasma proteins	rho	0.385	0.442	0.478	0.399	-0.333
	p	0.0429	0.0248	0.0135	0.0331	0.0896
Phospholipids	rho	-0.672	-0.581	-0.667	-0.589	0.602
	p	9.12e-05	0.00223	0.000201	0.000973	0.000893

## Discussion

In the present study we have characterized for the first time the metabolic fingerprint of PMNs obtained from morbid obese patients. The main metabolic change was a decrease in 3-hydroxyisovalerate and an increase in 2-aminobutyric acid in comparison with age-matched lean controls. In addition, this metabolic signature was strongly correlated with IR, BMI and waist circumference. These novel findings not only suggest that the metabolomics profile of PMNs could be a useful biomarker of IR, but also open up a new avenue for the research of the complex and bidirectional relationship between neutrophils and obesity.

3-hydroxyisovalerate, also named β-hydroxyisovalerate or 3-hydroxy-3-methylbutyric acid, is a final product of leucine catabolism, which is not further degraded and, therefore, can be contemplated as a useful biomarker. Leucine, isoleucine and valine are the three branched-chain amino acids (BCAAs) which are catabolized by mitochondrial dehydrogenase and branched-chain keto acid dehydrogenase (BCKADH) to fuel the Krebs cycle for ATP production. Recent studies have shown the positive association between increased circulating BCAAs and insulin resistance (IR) in obese or diabetic patients [24–27]. In addition, plasma BCCAs have been associated with visceral adipose tissue (VAT) [28]. A hypothesized mechanism linking increased levels of BCAAs and type 2 diabetes involved leucine-mediated activation of the mammalian target of rapamycin complex 1 (mTORC1), which resulted in the uncoupling of insulin signalling at an early stage [29]. In addition, defective BCAA catabolism might occur in obesity, leading to a further accumulation of BCAAs and toxic intermediates [30]. In fact, it seems more plausible to consider increased BCAA levels a biomarker of IR rather than themselves as being causative [29]. Insulin induces the expression and activity of BCKADH, the rate-limiting enzyme in the BCAA degradation pathway [31]. Therefore, the lower levels of 3-hydroxyisolvalerate detected in obese individuals in comparison with lean subjects indicate that a decreased catabolism of leucine occurs within the PMNs of obese individuals. In addition, the inverse relationship between 3-hydrovalerate and HOMA-IR suggest that an insulin signalling impairment could participate in the reduction of leucine catabolism. To the best of our knowledge, this is the first time that alterations in BCAA metabolism have been detected in the PMNs of obese subjects, as well as their association with IR.

2-aminoisobutyric acid, also called α-aminoisobutyric acid, is a non proteinogenic BCAA which results as the end product of pyrimidin metabolism. With the exception of a few bacteria, it is non-metabolisable, and therefore very useful in bioassays. At present, we do not have any robust explanation for the strong relationship between the increase of 2-aminoisobutyric in the PMN and the levels of HOMA-IR or BMI and waist circumference.

Overall, we have developed a tool for assessing the insulin resistance directly in PMNs. This could give us complementary information that is currently obtained by HOMA and might respresent a new strategy for measuring the direct effect of diets and drugs for the treatment of diabetes at cellular level.

The consequences of the metabolic changes observed in the neutrophils from obese patients require elucidation. Classically, neutrophils have been considered the “kamikaze” cells that arrive first at the site of injury and immolate themselves while killing the invading pathogens with a variety of mechanisms [32]. However, a growing body of evidence is challenging this view, suggesting that neutrophils may exert a more complex role interacting with other components of the innate and adaptive immune system [33]. Although the functional consequences of the metabolic abnormalities in PMNs here reported need to be investigated, it is possible that our findings are related with the increase of infectious diseases reported in obese patients (such as surgical-site infections, nosocomial infections, periodontitis and skin infections [34]. In addition, the potential effect of detected metabolic changes on the migration capacity of PMNs, favoring their deposition in visceral adipose tissue or the vascular wall, also need to be examined.

It is worth mentioning that the metabolic profile in plasma was different than that obtained in PMN, thus ruling out any significant interference of plasma on our results. In addition, the abnormalities detected in the plasma of obese patients, such as the increase of valine, were in agreement with previous reports [35], thus confirming the reliability of our methodology.

Our study has two main limitations. First, this is a pilot study which has been only validated in a small independent cohort and, therefore, further confirmation in larger studies are required. Second, as occurs in all cross-sectional studies a direct causal role between the metabolic changes of neutrophils and their functional impairment can not be established. However, rather than any type of causality our objective was to identify a metabolic signature of PMNs in the setting of morbid obesity and the underlying insulin resistance.

In conclusion, we have described an easy and reliable method to monitor the metabolic signature of obesity using PMNs cells. Our results suggest that 3-hydroxyisovalerate and 2-aminoisobutyric acid are key metabolic biomarkers of IR and anthropometric features of obesity. Finally, the methodology described could be used for monitoring the effect of diets and treatments, thus opening up a new avenue in the setting of precision medicine.

## Supporting information

S1 Table Identified metabolites and integration regions in the NMR spectrum. (PDF)

S2 Table Clinical data of patients. (PDF)

S3 Table Raw metabolite integration data from normal individuals. (PDF)

S4 Table Raw metabolite integration data from obese individuals. (PDF)

S5 Table Summary of anthropometrical and biochemical parameters of obese patients and healthy individuals from the validation study. (PDF)

S6 Table Normalized concentration values of selected metabolites from obese and normal individuals from the validation study. (PDF)

S1 Fig Score plot from PCA of obese and normal weight individuals. Input variables are age, the BMI, waist, HDL, LDL, TG glucose, insulin and HOMA-IR. R2X = 0.65, Q2 = 0.30.
(TIF)

S2 Fig Loading plot from PCA of obese and normal weight individuals. Input variables are age, the BMI, waist, HDL, LDL, TG glucose, insulin and HOMA-IR.
(TIF)

S3 Fig PCA distance to Model X. Plot of obese and normal weight individuals. Input variables are age, the BMI, waist, HDL, LDL, TG, glucose, insulin and HOMA-IR.
(TIF)

S4 Fig PCA analysis of the metabolic profile of PMN cells from obese patients and normal weight individuals. A) Score plot of components 1 and 2. B) Score plot of components 1 and 4. Principal Component 1: R2X = 0.37, Q2 = 0.28. Principal Component 2: R2X = 0.16, Q2 0.07. Principal Component 3: R2X = 0.13, Q2 0.18. Principal Component 4: R2X = 0.06, Q2 0.01.
(TIF)

S5 Fig Permutation test after 50 permutations performed with a PLS-DA model of the metabolic profile of PMN cells from samples of normal and obese patients. Intercepts: R2 = 0.0, 0.268 Q2 = 0.0, -0.247. PLS-DA model values: R2 = 0.812, Q2 = .0.547.
(TIF)

S6 Fig Permutation test after 50 permutations performed with the PLS analysis of the metabolic profile of PMN cells from samples of normal and obese patients versus the BMI value. Intercepts: R2 = 0.0, 0.295, Q2 = 0.0, -0.199. Model values: R2 = 0.709, Q2 = 0.410.
(TIF)

S7 Fig Loading plot from the PLS-DA of the metabolic profile of PMN cells from samples of normal and obese patients. (TIF)

S8 Fig PCA loading plot from metabolic profile of plasma from obese patients and normal weight controls. (TIF)
