# Supplementary material for: Metabolic fingerprint of insulin resistance in human polymorphonuclear leucocytes
Source: PLoS One. 2018 Jul 13;13(7):e0199351. doi: 10.1371/journal.pone.0199351 (PMC6044522; doi:10.1371/journal.pone.0199351)
Supplement: S1 Table — (PDF) [file pone.0199351.s001.pdf]

metabolite number	left limit (ppm)	right limit (ppm)	metabolite
1	1.47	1.477	2-aminoisobutyric acid
2	1.237	1.247	3-hydroxyisovalerate
3	1.888	1.947	acetate
4	2.224	2.244	acetoacetate
5	6.15	6.169	ADP
5	8.534	8.546	ADP
5+6	8.264	8.282	ADP+ATP
7	1.477	1.496	alanine
8	2.79	2.813	aspartate
8	2.822	2.841	aspartate
8	2.857	2.872	aspartate
6	6.12	6.15	ATP
6	8.546	8.555	ATP
6	8.574	8.588	ATP
5+6	4.495	4.54	ATP+ADP
5+6	4.595	4.634	ATP+ADP
9	2.506	2.519	b-alanine
9	2.519	2.53	b-alanine
10	2.641	2.656	citrate
10	2.656	2.667	citrate
11+12	2.976	3.044	creatine/phosphocreatine
11+12	3.922	3.948	creatine/phosphocreatine
13	8.451	8.467	formate
14+15+16	5.925	5.967	GDP/GTP/GMP
17	3.454	3.476	glucose
17	3.473	3.5	glucose
17	3.516	3.554	glucose
17	3.691	3.76	glucose
17	3.76	3.792	glucose
17	4.634	4.67	glucose
17	5.223	5.251	glucose
18	2.323	2.368	glutamate
18	2.048	2.1	glutamate
19	2.433	2.445	glutamine

19	2.445	2.47	glutamine
19	2.47	2.482	glutamine
18+19	2.1	2.136	glutamine/glutamate
18+19	2.136	2.147	glutamine/glutamate
20	3.631	3.669	glycerol
21	3.554	3.565	glycine
22	5.37	5.5	glycogen
23+24	3.219	3.23	GPC/PC
25	4.551	4.584	GSH
25+26	2.53	2.547	GSH+GSSG
25+26	2.547	2.559	GSH+GSSG
25+26	2.559	2.581	GSH+GSSG
25+26	2.918	2.932	GSH+GSSG
25+26	2.932	2.976	GSH+GSSG
27	6.09	6.12	inosine
28	1.005	1.027	isoleucine
29	1.313	1.346	lactate
29	4.084	4.143	lactate
30	0.951	0.982	leucine
31	3.353	3.368	methanol
32	2.719	2.74	methionine sulfoxide
33	2.628	2.641	methionine
34	2.287	2.323	methylacetoacetate
35	3.57	3.58	myo-inositol
36	2.028	2.048	N-acetylaspartate
37	1.506	1.556	N-Methyl-a-aminoisobutyric acid
38	1.788	1.803	ornitine
38	1.804	1.826	ornitine
24	3.204	3.212	PC
39	7.299	7.443	phenylalanine
40	8.603	8.621	purine
41	2.368	2.374	pyruvate
42	3.58	3.631	sarcosine
43	2.381	2.4	succinate
44	3.252	3.288	taurine

44	3.411	3.448	taurine
45	7.765	7.802	tryptophane
46	6.894	6.926	tyrosine
46	7.184	7.218	tyrosine
47	5.967	6.021	UDP-NAG
48	0.982	1.005	valine
48	1.027	1.058	valine
