# Supplementary material for: Metabolic fingerprint of insulin resistance in human polymorphonuclear leucocytes
Source: PLoS One. 2018 Jul 13;13(7):e0199351. doi: 10.1371/journal.pone.0199351 (PMC6044522; doi:10.1371/journal.pone.0199351)
Supplement: S2 Table — (PDF) [file pone.0199351.s002.pdf]

patient	weight	age	IMC	waist	glucose	HDL	LDL	TG	insulin	HOMA	LDL/HDL	Log(TG)	Log(TG)/HDL
1	normal	38	24.61	88	133	40	175	133	14.6	4.79	4.38	2.12	0.053
2	normal	58	24.55	92	120	32	139	216	6.9	2.04	4.34	2.33	0.073
3	normal	47	23.73	87	104	46	131	70	9.6	2.47	2.85	1.85	0.04
4	normal	52	21.56	80	95	62	164	90	4.9	1.15	2.65	1.95	0.032
5	normal	55	23.69	85	96	54	158	100	9.7	2.3	2.93	2	0.037
6	normal	64	24	106	94	50	106	148	8.5	1.97	2.12	2.17	0.043
7	normal	62	24.9	96	106	55	143	132	9.6	2.51	2.6	2.12	0.039
8	normal	35	21.23	74	99	48	112	73	5.6	1.37	2.33	1.86	0.039
9	normal	41	24.95	88	100	38	110	158	6.7	1.65	2.89	2.2	0.058
10	normal	35	18.54	73	81	42	121	143	10.2	2.04	2.88	2.16	0.051
11	normal	21	24.72	86	72	NA	47	68	NA	NA	NA	1.83	NA
12	normal	25	22.95	NA	83	33	60	91	10.4	2.13	1.82	1.96	0.059
13	normal	34	20.17	86	89	61	91	46	6.5	1.43	1.49	1.66	0.027
14	normal	42	23.51	88	84	39	112	80	NA	NA	2.87	1.9	0.049
15	normal	26	16.65	72	88	34	48	105	4.2	0.91	1.41	2.02	0.059
16	normal	23	19.33	82	87	41	101	84	6.5	1.4	2.46	1.92	0.047
17	normal	38	21.25	NA	80	49	104	61	NA	NA	2.12	1.79	0.036
18	obese	31	37.98	117	80	NA	NA	NA	9.6	1.9	NA	NA	NA
19	obese	34	37.96	119	94	34	114	97	14.2	3.3	3.35	1.99	0.058
20	obese	37	39.8	110	77	32	122	123	12.1	2.3	3.81	2.09	0.065
21	obese	38	57.31	150	95	27	123	130	37.5	8.8	4.56	2.11	0.078
22	obese	35	42.55	143	74	41	143	157	21.5	3.93	3.49	2.2	0.054
23	obese	35	56.2	150	98	31	110	284	24.3	5.88	3.55	2.45	0.079
24	obese	55	43.05	114	75	41	133	170	30.5	5.65	3.24	2.23	0.054
25	obese	29	44.14	113	95	42	102	69	14.9	3.5	2.43	1.84	0.044
26	obese	23	39.58	NA	94	41	136	233	14	3.25	3.32	2.37	0.058
27	obese	40	40.22	134	112	29	47	146	26.4	7.3	1.62	2.16	0.075
28	obese	45	51.99	138	106	46	94	159	10.3	2.7	2.04	2.2	0.048
29	obese	49	45.73	137	111	38	62	86	11.8	3.23	1.63	1.93	0.051
30	obese	46	54.82	122	103	40	189	185	23.4	5.95	4.73	2.27	0.057
31	obese	44	47.1	150	100	39	119	120	19	4.69	3.05	2.08	0.053
32	obese	55	45.88	145	118	37	65	90	22.3	6.5	1.76	1.95	0.053
33	obese	54	40.64	120	102	32	105	90	9.3	2.34	3.28	1.95	0.061
34	obese	42	48.5	109	100	43	121	76	18.1	4.47	2.81	1.88	0.044
