# Supplementary material for: Metabolic fingerprint of insulin resistance in human polymorphonuclear leucocytes
Source: PLoS One. 2018 Jul 13;13(7):e0199351. doi: 10.1371/journal.pone.0199351 (PMC6044522; doi:10.1371/journal.pone.0199351)
Supplement: S3 Table — (PDF) [file pone.0199351.s003.pdf]

		Patient number	1	2	3	4	5	6	7	8	9	10	11	12	13	14	15	16	17	mean	SEM
left limit (ppm)	right limit (ppm)	metabolite	normal	normal	normal	normal	normal	normal	normal	normal	normal	normal	normal	normal	normal	normal	normal	normal	normal	mean normal	SEM normal
1.47	1.477	2-aminoisobutyric acid	3.201	2.910	3.084	2.902	2.871	2.733	2.795	2.679	3.694	2.444	2.274	2.533	2.424	2.421	2.395	2.702	1.819	2.699	0.102
1.237	1.247	3-hydroxyisovalerate	33.969	15.152	28.041	24.347	17.282	26.709	16.603	14.688	24.414	10.422	8.438	8.953	13.889	15.343	17.526	12.205	7.641	17.390	1.843
1.888	1.947	acetate	22.332	21.307	22.798	23.796	19.554	20.406	19.744	20.677	25.424	18.621	17.624	18.407	19.601	18.786	17.451	19.911	14.551	20.058	0.629
2.224	2.244	acetoacetate	8.235	5.272	6.976	6.572	5.157	7.034	4.826	4.834	6.614	4.080	4.141	4.140	4.707	4.837	5.321	4.599	3.376	5.336	0.316
6.15	6.169	ADP	1.321	1.653	1.382	1.248	1.695	1.523	0.921	1.519	1.542	1.672	0.773	1.877	1.525	1.491	2.223	1.648	1.975	1.529	0.085
8.534	8.546	ADP	1.475	0.933	1.178	1.019	0.832	1.301	0.577	0.714	1.064	0.653	0.449	0.912	0.771	0.874	1.101	0.772	1.063	0.923	0.064
8.264	8.282	ADP+ATP	2.204	1.644	1.851	1.776	1.557	2.309	1.128	1.323	1.790	1.104	0.969	1.620	1.588	1.590	2.467	1.639	1.771	1.666	0.099
1.477	1.496	alanine	10.658	10.665	10.017	10.170	10.415	9.462	10.537	11.286	11.450	11.706	8.940	9.538	9.276	10.460	8.572	11.768	8.398	10.195	0.253
2.79	2.813	aspartate	3.162	2.448	2.651	2.646	2.450	2.690	2.060	2.209	2.840	2.026	2.215	2.615	2.644	2.595	2.435	2.961	2.340	2.529	0.074
2.822	2.841	aspartate	2.231	1.812	2.061	2.052	1.881	2.216	1.600	1.608	2.083	1.553	1.753	1.845	1.919	1.798	1.838	2.111	1.668	1.884	0.052
2.857	2.872	aspartate	1.852	1.561	1.823	1.868	1.605	1.520	1.603	1.408	1.888	1.261	1.420	1.215	1.484	1.505	1.327	1.311	0.937	1.505	0.063
6.12	6.15	ATP	1.891	1.672	1.829	1.537	1.841	1.956	1.186	1.701	2.040	1.602	1.006	1.705	1.483	1.702	2.447	1.757	1.773	1.713	0.078
8.546	8.555	ATP	0.943	0.596	0.889	0.702	0.586	1.010	0.476	0.525	0.845	0.449	0.304	0.722	0.595	0.591	0.756	0.467	0.884	0.667	0.048
8.574	8.588	ATP	1.542	1.074	1.443	1.312	1.357	1.226	0.853	1.349	1.424	1.741	0.549	0.790	0.820	0.892	0.882	0.974	0.632	1.109	0.083
4.495	4.54	ATP+ADP	3.263	5.871	3.803	5.486	5.189	4.267	3.847	5.464	3.967	6.432	6.160	7.625	6.192	5.852	7.075	6.739	6.161	5.494	0.307
4.595	4.634	ATP+ADP	6.054	5.961	3.286	5.732	4.136	4.839	4.146	3.940	3.577	4.439	4.789	5.758	5.741	3.718	5.115	4.804	5.936	4.822	0.224
2.506	2.519	b-alanine	2.079	1.568	1.992	1.479	1.310	1.664	1.143	1.242	1.603	1.136	1.115	1.015	1.130	1.193	1.303	1.278	1.061	1.371	0.077
2.519	2.53	b-alanine	1.995	1.444	1.785	1.321	1.287	1.594	1.209	1.152	1.546	1.089	1.031	1.048	1.029	1.110	1.270	1.195	1.138	1.308	0.068
2.641	2.656	citrate	2.763	2.556	2.479	2.533	2.604	2.556	2.126	2.626	2.643	2.462	2.633	2.190	2.627	2.753	3.056	2.841	2.912	2.609	0.056
2.656	2.667	citrate	2.116	1.879	1.787	1.923	1.988	1.887	1.406	1.846	1.869	1.920	1.953	1.832	2.022	2.071	2.149	2.256	2.256	1.951	0.049
2.976	3.044	creatine/phosphocreatine	13.275	23.763	16.953	24.211	22.994	16.214	21.798	21.086	21.287	22.373	21.312	23.695	22.964	21.976	19.793	20.371	18.089	20.715	0.729
3.922	3.948	creatine/phosphocreatine	4.553	6.724	5.196	6.726	7.199	6.183	10.360	8.924	5.620	8.901	8.993	9.190	8.421	9.178	7.483	9.314	9.605	7.798	0.419
8.451	8.467	formate	2.679	1.159	2.058	1.787	1.296	2.100	1.162	1.075	1.799	0.813	0.668	0.610	1.066	1.108	1.250	0.903	0.596	1.302	0.143
5.925	5.967	GDP/GTP/GMP	2.699	1.961	1.971	2.054	1.720	2.342	1.186	1.657	2.260	1.506	1.074	1.155	1.668	1.376	2.159	1.396	1.409	1.741	0.113
3.411	3.448	glucose	12.671	31.742	15.822	12.774	32.628	24.533	11.263	25.978	18.458	53.955	55.842	35.546	24.521	36.784	31.506	28.121	71.015	30.774	4.006
3.454	3.476	glucose	2.322	1.763	2.264	1.900	1.735	2.247	2.677	2.028	1.902	1.934	2.193	1.958	2.344	1.873	2.055	1.615	2.141	2.056	0.065
3.473	3.5	glucose	2.901	2.213	2.875	2.379	2.167	2.672	4.124	2.847	2.252	2.232	2.734	2.701	3.030	2.474	2.640	2.339	2.745	2.666	0.113
3.516	3.554	glucose	6.204	4.390	5.671	5.043	5.651	5.723	7.441	5.170	5.138	4.771	5.343	6.049	5.360	5.302	4.883	6.148	5.728	5.530	0.169
3.691	3.76	glucose	42.340	28.181	27.890	29.523	24.648	29.061	38.717	31.247	27.645	31.191	26.978	31.699	27.783	33.489	27.388	27.096	26.823	30.100	1.099
3.76	3.792	glucose	10.317	15.770	11.056	12.665	17.368	14.440	18.264	19.015	12.671	21.291	19.505	20.180	17.656	21.543	20.280	18.739	24.235	17.353	0.961
4.634	4.67	glucose	6.447	4.680	3.121	4.366	3.065	3.124	3.750	3.056	2.628	3.161	4.007	3.373	3.855	1.921	3.406	3.795	3.472	3.602	0.237
5.223	5.251	glucose	2.008	0.878	0.781	0.990	0.816	1.467	0.657	0.821	0.759	0.981	1.267	0.842	1.070	0.658	0.746	1.004	1.112	0.992	0.082
2.323	2.368	glutamate	12.875	12.976	12.059	11.254	14.067	12.842	9.793	11.870	12.273	12.935	11.515	15.769	12.850	14.733	11.654	17.701	14.297	13.027	0.453
2.048	2.1	glutamate	17.084	23.615	19.607	25.668	23.218	21.458	20.287	21.814	22.152	22.989	22.164	30.989	25.747	28.676	24.415	26.997	24.797	23.628	0.824
2.433	2.445	glutamine	2.434	1.889	2.304	1.850	1.628	2.087	1.649	1.773	1.953	1.690	1.677	1.369	1.468	1.725	1.575	1.455	1.368	1.759	0.074
2.445	2.47	glutamine	5.708	4.834	5.445	4.426	4.255	5.218	3.635	4.408	4.872	4.735	4.152	3.264	3.467	4.278	4.075	3.506	3.849	4.360	0.172
2.47	2.482	glutamine	2.304	1.629	1.935	1.546	1.439	1.872	1.253	1.423	1.770	1.346	1.285	1.037	1.187	1.292	1.316	1.157	1.073	1.463	0.083
3.631	3.669	glycerol	9.474	9.549	10.041	11.058	9.330	9.781	18.400	13.327	10.499	9.140	11.559	11.946	11.764	13.338	10.613	9.643	10.251	11.160	0.552
3.554	3.565	glycine	3.448	3.080	3.436	2.645	3.630	3.129	3.942	3.275	3.221	3.702	3.653	2.856	3.026	3.685	3.690	3.561	4.332	3.430	0.101
5.37	5.5	glycogen	2.958	3.117	4.745	2.664	2.247	4.546	4.904	3.912	3.466	2.484	4.076	2.715	2.463	3.199	3.352	2.091	2.127	3.239	0.222
3.219	3.23	GPC/PC	4.303	5.851	3.379	4.572	9.526	5.533	3.780	5.740	4.777	9.716	5.014	10.560	7.414	8.266	5.950	13.357	9.901	6.920	0.688
2.932	2.976	GSH	5.286	4.958	4.977	4.935	6.209	6.155	3.702	5.591	5.410	6.096	7.227	4.298	5.199	4.932	7.755	4.406	6.238	5.493	0.251
4.551	4.584	GSH	3.238	3.766	2.410	4.076	3.287	3.475	2.738	3.385	2.566	4.150	4.495	4.078	4.477	3.552	4.642	3.359	4.513	3.659	0.169
2.53	2.547	GSH+GSSG	2.933	2.682	2.906	2.127	2.619	2.806	1.650	2.261	2.726	2.489	2.121	1.957	1.900	2.373	2.764	2.379	2.815	2.442	0.094
2.547	2.559	GSH+GSSG	2.417	2.447	2.228	1.862	2.540	2.609	1.740	2.391	2.212	2.762	2.273	1.864	2.083	2.346	3.115	2.132	3.193	2.365	0.097
2.559	2.581	GSH+GSSG	4.074	3.632	3.854	3.452	4.136	4.176	2.498	3.795	3.806	4.379	4.561	4.208	5.480	3.773	4.853	3.282	5.158	4.066	0.173
2.918	2.932	GSH+GSSG	1.326	1.008	1.364	1.182	1.160	1.273	0.885	0.999	1.175	0.899	1.273	0.744	0.985	0.882	1.267	0.806	0.882	1.065	0.048
2.932	2.976	GSH+GSSG	1.760	1.087	1.439	1.115	1.138	1.476	1.015	1.065	1.453	0.966	0.624	0.665	0.967	1.192	1.653	1.047	1.257	1.172	0.075
6.09	6.12	inosine	0.740	0.513	0.740	0.561	0.565	0.741	0.442	0.485	0.658	0.532	0.304	0.322	0.480	0.588	0.729	0.555	0.574	0.561	0.033
1.005	1.027	isoleucine	4.139	4.412	3.723	4.445	4.158	3.771	4.422	4.277	4.019	4.689	4.849	4.003	4.155	4.395	3.938	4.932	3.705	4.237	0.089
1.313	1.346	lactate	25.791	45.656	29.912	16.879	29.338	32.616	22.287	39.150	28.582	47.896	74.751	21.351	33.789	23.780	25.255	37.778	60.890	35.041	3.660
4.084	4.143	lactate	8.242	14.246	10.010	10.505	11.089	11.187	13.201	14.054	10.643	14.844	22.139	12.845	13.741	12.816	11.581	14.003	17.498	13.097	0.776
0.951	0.982	leucine	11.065	21.401	14.342	18.995	21.350	15.552	21.165	21.465	18.014	24.381	20.031	22.114	22.238	22.584	20.038	25.632	19.688	20.003	0.881
3.353	3.368	methanol	4.541	4.836	3.957	4.092	3.810	4.362	3.099	8.357	4.680	2.798	2.264	2.845	4.941	4.395	3.875	2.981	2.832	4.039	0.336
2.628	2.641	methionine	2.251	1.914	1.988	2.002	1.786	2.119	1.453	1.762	2.019	1.680	1.794	1.530	1.777	1.756	1.963	1.939	1.677	1.848	0.050
2.287	2.323	methylacetoacetate	10.244	9.902	9.782	10.483	9.640	9.246	8.499	9.470	9.988	8.947	8.419	10.423	9.558	9.441	9.511	9.881	8.094	9.501	0.167
3.57	3.58	myo-inositol	2.180	1.581	2.232	1.918	1.738	1.832	2.980	1.960	1.863	1.736	1.881	1.979	1.955	2.077	1.918	1.663	1.951	1.967	0.075
2.028	2.048	N-acetylaspartate	7.761	11.655	9.784	13.927	11.000	10.574	11.033	10.833	10.674	10.990	10.804	17.344	14.278	15.533	12.930	12.604	12.148	11.992	0.555
1.788	1.803	ornitine	8.310	5.759	7.884	7.213	5.375	7.007	5.373	5.412	7.671	4.048	4.252	4.277	5.153	4.682	4.845	4.485	3.192	5.585	0.365
1.804	1.826	ornitine	8.832	7.255	8.711	8.733	6.562	7.476	7.282	7.178	9.200	5.153	5.722	5.887	6.485	5.690	5.593	5.654	4.198	6.801	0.353
3.204	3.212	PC	1.254	2.069	1.606	1.807	2.568	1.709	1.769	1.761	1.808	2.003	2.575	1.964	1.705	1.937	1.605	2.033	1.898	1.887	0.079
7.299	7.443	phenylalanine	17.624	13.285	16.836	15.609	13.818	16.525	12.067	12.417	16.020	12.211	11.215	10.604	12.354	13.029	14.293	12.764	9.637	13.548	0.557
8.603	8.621	purine	2.277	1.875	2.266	1.972	1.963	2.663	1.339	1.732	2.156	1.220	1.079	1.778	1.809	1.861	3.239	2.141	1.728	1.947	0.125
2.368	2.374	pyruvate	1.736	1.690	1.587	1.504	1.743	1.690	1.414	1.661	1.568	1.693	1.468	2.090	1.733	1.842	1.515	2.369	1.916	1.719	0.058
3.58	3.631	sarcosine	9.110	9.179	9.424	10.322	10.287	10.338	17.919	12.399	9.281	10.263	11.121	13.567	11.232	12.780	10.503	12.128	11.985	11.285	0.525
2.381	2.4	succinate	6.665	5.021	6.208	5.644	4.847	5.944	4.125	4.407	5.561	4.031	3.799	4.447	4.350	4.482	4.848	4.420	3.598	4.847	0.213
3.252	3.288	taurine	10.846	34.110	16.260	11.159	34.701	24.946	10.385	26.980	19.253	59.245	58.072	35.986	23.946	36.650	31.922	30.466	72.838	31.633	4.304
3.411	3.448	taurine	1.738	5.092	3.658	4.897	4.905	3.573	4.527	4.724	4.088	5.411	4.649	6.667	5.370	5.839	6.176	5.312	5.727	4.844	0.279
7.765	7.802	tryptophane	4.616	2.229	3.955	3.403	2.444	4.053	2.281	2.208	3.224	1.610	1.416	1.275	1.965	2.150	2.552	1.715	1.184	2.487	0.248
6.894	6.926	tyrosine	2.746	1.873	2.360	2.227	2.028	2.595	1.668	2.056	2.281	1.985	1.813	1.443	1.778	2.019	2.005	1.886	1.468	2.014	0.085
7.184	7.218	tyrosine	3.527	2.559	3.063	2.835	2.669	3.235	2.407	2.567	3.014	2.643	2.596	1.940	2.471	2.644	2.667	2.665	2.134	2.684	0.092
5.967	6.021	UDP-NAG	3.947	2.705	3.076	3.031	2.832	3.688	1.893	2.404	3.123	2.335	1.699	2.038	2.407	2.466	2.860	2.662	2.238	2.671	0.144
0.982	1.005	valine	4.861	5.739	5.257	5.470	6.164	5.201	5.926	5.742	5.500	6.479	6.234	5.491	6.258	5.776	5.704	6.939	5.187	5.761	0.129
1.027	1.058	valine	5.618	5.082	4.946	5.198	4.956	5.345	5.263	5.057	5.231	5.470	6.032	4.107	4.554	5.355	4.583	6.049	4.223	5.122	0.132
