# Supplementary material for: Metabolic fingerprint of insulin resistance in human polymorphonuclear leucocytes
Source: PLoS One. 2018 Jul 13;13(7):e0199351. doi: 10.1371/journal.pone.0199351 (PMC6044522; doi:10.1371/journal.pone.0199351)
Supplement: S4 Table — (PDF) [file pone.0199351.s004.pdf]

		Patient number	18	19	20	21	22	23	24	25	26	27	28	29	30	31	32	33	34		
left limit (ppm)	right limit (ppm)	metabolite	obese	obese	obese	obese	obese	obese	obese	obese	obese	obese	obese	obese	obese	obese	obese	obese	obese	mean obese	SEM obese
1.47	1.477	2-aminoisobutyric acid	4.313	4.043	4.983	5.066	4.189	3.664	4.710	4.835	4.573	1.930	4.301	3.005	3.286	5.484	3.608	5.103	4.808	4.229	0.202
1.237	1.247	3-hydroxyisovalerate	9.605	7.546	9.821	9.360	7.263	6.192	8.971	7.347	8.417	2.995	7.905	4.941	5.712	12.385	6.802	10.831	9.043	7.949	0.508
1.888	1.947	acetate	27.208	28.379	30.851	28.060	28.503	23.520	28.239	27.838	28.825	21.079	26.824	19.938	21.193	32.066	21.395	27.161	30.066	26.538	0.828
2.224	2.244	acetoacetate	4.949	4.570	5.683	5.588	4.838	4.227	4.794	5.011	4.768	2.259	4.817	3.694	3.931	6.453	4.034	5.197	5.273	4.711	0.208
6.15	6.169	ADP	1.154	1.491	1.335	1.778	1.221	1.860	1.211	0.987	0.968	1.268	1.328	1.549	2.039	2.124	2.432	1.311	1.231	1.488	0.094
8.534	8.546	ADP	1.084	0.961	1.010	1.120	0.730	0.780	0.906	0.725	0.813	0.691	0.748	0.750	1.033	1.524	1.049	1.057	0.880	0.933	0.047
8.264	8.282	ADP+ATP	1.671	1.596	1.725	1.837	1.361	1.680	1.587	1.324	1.445	1.154	1.683	1.304	1.797	2.442	1.811	1.898	1.476	1.635	0.066
1.477	1.496	alanine	7.502	9.675	9.869	11.501	10.631	9.240	11.102	14.089	12.076	5.467	10.512	8.379	9.544	8.553	8.934	9.402	11.296	9.869	0.435
2.79	2.813	aspartate	2.247	2.985	2.763	2.562	2.842	2.163	2.717	2.618	2.472	1.309	2.727	2.040	2.001	3.175	2.650	3.020	2.682	2.528	0.103
2.822	2.841	aspartate	1.899	2.189	2.108	2.033	2.087	1.737	1.981	2.014	1.923	0.930	2.207	1.562	1.450	2.376	1.917	2.232	2.073	1.925	0.078
2.857	2.872	aspartate	1.546	1.766	1.871	1.444	1.743	1.348	1.722	1.796	1.903	0.745	1.826	1.182	1.227	1.796	1.210	1.782	1.584	1.558	0.072
6.12	6.15	ATP	1.720	1.699	1.534	2.093	1.482	2.073	1.577	1.430	1.192	1.266	1.854	1.507	1.831	2.429	2.248	1.686	1.452	1.710	0.077
8.546	8.555	ATP	0.658	0.691	0.631	0.780	0.558	0.593	0.713	0.538	0.653	0.446	0.543	0.495	0.683	1.031	0.623	0.840	0.663	0.655	0.031
8.574	8.588	ATP	1.121	0.950	1.334	1.411	1.049	0.969	0.998	0.903	0.982	0.545	0.950	1.068	1.457	1.485	1.923	1.135	0.916	1.129	0.070
4.495	4.54	ATP+ADP	3.165	6.122	7.983	5.103	5.524	5.509	3.920	4.927	4.284	4.514	4.915	6.545	7.969	4.259	6.948	5.006	3.544	5.308	0.316
4.595	4.634	ATP+ADP	1.884	5.224	6.469	3.697	5.130	3.737	4.353	4.503	3.523	3.961	3.486	3.893	7.193	4.760	5.237	3.657	5.319	4.472	0.278
2.506	2.519	b-alanine	2.081	1.627	1.973	2.237	1.640	1.397	1.849	1.755	1.690	0.853	1.852	1.322	1.214	2.620	1.487	1.865	1.723	1.717	0.091
2.519	2.53	b-alanine	1.737	1.484	1.848	1.881	1.566	1.323	1.635	1.550	1.513	0.888	1.604	1.241	1.141	2.330	1.388	1.609	1.624	1.551	0.072
2.641	2.656	citrate	2.123	2.507	2.679	2.420	2.530	2.593	2.436	2.506	2.504	1.612	2.448	2.524	2.579	2.916	2.535	2.433	2.682	2.472	0.061
2.656	2.667	citrate	1.538	2.007	1.836	1.847	1.952	1.897	1.950	1.859	1.877	1.217	1.900	1.777	1.920	2.139	2.015	1.919	2.011	1.862	0.047
2.976	3.044	creatine/phosphocreatine	12.161	24.799	23.665	16.685	27.128	22.735	23.218	26.776	26.812	13.774	24.870	18.102	22.635	14.633	18.873	20.828	23.757	21.262	1.062
3.922	3.948	creatine/phosphocreatine	4.995	8.438	7.295	6.609	8.108	10.364	7.738	8.585	8.984	8.237	8.096	10.849	8.754	5.142	8.036	8.253	8.383	8.051	0.334
8.451	8.467	formate	1.848	1.322	1.761	1.746	1.397	1.173	1.776	1.312	1.595	0.625	1.421	0.945	1.056	2.572	1.282	2.097	1.677	1.506	0.103
5.925	5.967	GDP/GTP/GMP	2.117	1.858	2.687	2.182	1.901	1.668	1.978	1.551	1.823	1.195	1.631	1.360	1.872	2.952	1.953	1.855	1.742	1.901	0.096
3.411	3.448	glucose	32.022	19.729	13.529	30.493	16.609	37.370	16.543	24.569	19.581	30.368	22.729	25.625	51.249	36.029	65.954	16.565	22.559	28.325	3.049
3.454	3.476	glucose	18.155	1.829	2.380	2.220	1.487	2.372	2.095	1.941	1.837	1.542	1.880	4.353	2.288	2.396	2.166	2.570	2.319	3.166	0.875
3.473	3.5	glucose	26.953	2.558	2.794	2.579	2.253	3.099	2.783	2.555	2.565	2.002	2.529	6.553	2.632	2.944	2.627	3.328	2.975	4.337	1.322
3.516	3.554	glucose	16.671	5.015	4.836	4.636	4.436	4.910	4.832	4.820	4.741	3.347	5.078	8.387	4.154	5.117	4.844	5.687	4.797	5.665	0.673
3.691	3.76	glucose	54.103	25.579	25.913	19.082	21.313	24.586	22.938	23.312	28.286	267.166	21.975	43.124	25.039	19.507	19.698	26.936	24.528	40.770	13.197
3.76	3.792	glucose	20.235	15.509	13.749	13.141	13.783	19.411	13.578	15.121	16.585	18.832	15.375	24.684	20.814	11.539	19.426	14.364	15.713	16.580	0.772
4.634	4.67	glucose	18.371	3.467	2.970	2.454	4.143	2.785	3.854	3.702	2.225	2.412	3.065	3.762	4.098	4.704	4.298	2.928	5.422	4.392	0.828
5.223	5.251	glucose	10.930	0.881	1.080	0.835	1.305	1.149	1.311	0.911	0.960	0.772	0.909	1.773	1.002	1.430	1.000	1.069	1.386	1.688	0.536
2.323	2.368	glutamate	10.168	14.684	11.990	13.518	12.932	11.154	12.889	13.324	11.888	8.569	14.521	12.862	12.136	12.015	12.771	11.722	12.744	12.346	0.331
2.048	2.1	glutamate	18.365	29.384	25.886	20.416	26.314	26.631	23.612	25.747	28.346	17.475	25.704	24.667	26.632	20.090	21.612	22.743	26.763	24.141	0.779
2.433	2.445	glutamine	1.916	1.770	1.818	2.062	1.843	1.577	1.855	1.976	1.855	0.933	1.906	1.333	1.508	2.634	1.748	1.780	1.920	1.790	0.078
2.445	2.47	glutamine	4.737	4.216	4.770	5.377	4.169	4.218	4.369	4.788	4.585	2.610	4.557	3.529	4.204	6.702	5.039	4.683	5.030	4.564	0.188
2.47	2.482	glutamine	1.728	1.433	1.581	1.939	1.379	1.283	1.442	1.613	1.514	0.763	1.582	1.129	1.167	2.320	1.529	1.571	1.610	1.505	0.075
3.631	3.669	glycerol	8.366	11.867	12.227	9.259	9.504	10.882	10.542	10.598	13.269	8.323	10.463	14.409	11.793	9.823	9.138	12.678	11.688	10.872	0.386
3.554	3.565	glycine	2.466	2.555	3.024	3.363	2.626	2.962	3.039	2.788	2.809	2.415	2.869	4.798	3.078	2.838	3.250	3.052	2.802	2.984	0.119
5.37	5.5	glycogen	4.893	3.653	4.272	5.126	2.605	2.936	3.283	3.010	3.494	2.782	3.872	4.777	2.289	4.799	2.634	3.967	2.095	3.558	0.216
3.219	3.23	GPC/PC	3.389	8.149	4.720	6.100	8.392	6.644	6.961	6.366	5.743	4.858	7.762	8.121	7.410	4.293	8.876	5.589	5.229	6.388	0.356
2.932	2.976	GSH	3.968	4.462	5.549	5.651	4.934	5.911	4.943	4.842	4.638	3.621	5.882	5.283	7.398	6.563	7.047	5.533	5.164	5.376	0.225
4.551	4.584	GSH	1.807	3.660	5.629	3.008	3.642	3.350	2.811	3.080	3.016	3.158	3.103	3.717	5.996	3.060	4.358	3.284	2.868	3.503	0.227
2.53	2.547	GSH+GSSG	2.637	2.336	2.631	3.035	2.339	2.563	2.473	2.576	2.304	1.869	2.645	2.196	2.222	3.524	2.769	2.395	2.505	2.531	0.082
2.547	2.559	GSH+GSSG	1.939	2.114	2.084	2.662	2.084	2.569	2.059	2.128	2.175	1.967	2.386	2.576	2.736	3.158	3.161	2.176	2.229	2.365	0.086
2.559	2.581	GSH+GSSG	3.121	3.665	3.545	4.275	3.647	4.151	3.318	3.402	3.297	5.664	4.003	7.940	4.401	4.908	4.565	3.606	3.712	4.189	0.261
2.918	2.932	GSH+GSSG	1.091	1.123	1.462	1.075	1.132	1.034	1.156	1.094	1.139	0.555	1.153	0.980	1.122	1.622	1.103	1.326	1.095	1.133	0.049
2.932	2.976	GSH+GSSG	1.464	1.148	1.237	1.390	1.198	1.695	1.523	1.512	1.715	1.111	1.342	0.935	0.946	2.038	1.098	1.678	1.976	1.412	0.074
6.09	6.12	inosine	0.691	0.446	0.759	0.622	0.533	0.758	0.686	0.637	0.704	0.515	0.531	0.483	0.518	0.768	0.591	0.825	0.733	0.635	0.026
1.005	1.027	isoleucine	3.140	4.645	4.728	3.956	5.218	4.350	5.260	5.734	5.596	2.798	4.717	3.533	4.297	3.870	3.666	5.168	4.816	4.441	0.190
1.313	1.346	lactate	16.436	17.260	20.450	36.390	26.179	51.004	33.802	14.977	15.956	72.387	34.181	34.966	25.596	16.251	44.547	29.078	18.180	29.861	3.433
4.084	4.143	lactate	7.532	11.290	12.856	11.713	12.186	16.352	12.604	10.593	11.580	18.588	12.752	16.999	12.528	6.954	13.861	12.116	9.583	12.358	0.672
0.951	0.982	leucine	10.995	22.953	20.866	18.473	27.270	22.176	25.554	28.079	25.992	13.516	23.311	19.246	22.871	12.356	19.306	23.304	23.201	21.145	1.121
3.353	3.368	methanol	6.021	5.186	5.767	4.836	4.242	4.159	4.931	4.087	5.052	2.484	4.791	4.919	6.526	7.371	6.604	6.473	5.446	5.229	0.263
2.628	2.641	methionine	1.651	1.849	2.031	1.920	1.848	1.801	1.852	1.825	1.846	1.061	1.788	1.626	1.836	2.286	1.674	1.763	1.930	1.799	0.054
2.287	2.323	methylacetoacetate	8.193	11.250	11.318	11.572	10.731	9.543	10.282	11.632	10.409	6.051	10.716	8.678	10.099	10.109	8.968	9.732	11.085	10.022	0.321
3.57	3.58	myo-inositol	1.499	1.640	1.961	1.636	1.582	1.760	1.790	1.616	1.906	1.344	1.756	4.140	1.600	1.746	1.352	2.179	1.835	1.844	0.140
2.028	2.048	N-acetylaspartate	8.773	15.478	13.025	9.520	13.299	14.149	11.931	13.217	15.892	9.376	12.952	12.799	14.446	9.598	10.418	11.725	14.005	12.388	0.490
1.788	1.803	ornitine	5.557	5.446	5.798	5.785	5.856	4.151	5.191	4.880	5.383	2.276	5.324	3.788	3.626	7.159	3.673	4.785	5.463	4.949	0.256
1.804	1.826	ornitine	7.366	7.785	8.075	8.180	8.209	5.804	7.365	7.094	7.307	3.465	7.387	5.572	5.116	9.184	4.943	6.173	7.675	6.865	0.328
3.204	3.212	PC	1.205	2.028	1.736	1.694	2.262	1.957	2.442	2.082	2.045	1.636	2.116	1.736	1.570	1.476	1.980	2.060	1.698	1.866	0.069
7.299	7.443	phenylalanine	13.100	13.263	16.314	14.214	14.208	13.637	15.537	14.536	15.173	7.382	14.113	11.031	12.486	16.577	12.661	17.964	14.661	13.933	0.531
8.603	8.621	purine	1.668	1.863	2.170	2.256	1.549	2.206	1.677	1.427	1.449	1.227	1.936	1.461	1.797	2.743	2.279	1.955	1.720	1.846	0.087
2.368	2.374	pyruvate	1.322	1.903	1.602	1.741	1.626	1.454	1.641	1.643	1.492	1.147	1.854	1.698	1.614	1.697	1.673	1.590	1.739	1.614	0.041
3.58	3.631	sarcosine	7.256	11.066	9.875	9.048	9.886	11.346	10.271	10.548	11.935	10.225	10.342	15.551	10.512	8.682	9.475	10.838	10.518	10.434	0.381
2.381	2.4	succinate	5.013	5.120	5.370	5.516	4.904	4.431	4.984	5.134	4.835	2.520	5.246	3.854	4.265	6.287	4.489	5.310	5.455	4.867	0.184
3.252	3.288	taurine	21.791	19.471	13.021	31.364	17.609	37.775	16.528	25.483	18.831	32.370	23.398	25.844	52.456	36.363	67.278	16.503	21.883	28.116	3.161
3.411	3.448	taurine	4.000	6.813	6.433	4.921	5.910	6.650	5.371	6.305	6.377	4.584	6.259	5.885	6.373	4.260	5.352	5.960	5.554	5.706	0.189
7.765	7.802	tryptophane	3.423	2.308	3.305	3.012	2.154	2.050	2.707	2.239	2.706	1.010	2.401	1.534	1.818	3.943	2.255	3.736	2.723	2.548	0.173
6.894	6.926	tyrosine	2.262	1.936	2.152	2.227	1.957	2.153	2.563	2.354	2.608	1.221	2.114	1.699	1.844	2.387	2.158	2.927	2.438	2.177	0.087
7.184	7.218	tyrosine	2.352	2.368	3.018	2.662	2.721	2.725	3.335	3.100	3.247	1.570	2.609	2.153	2.526	3.308	2.545	3.422	3.237	2.759	0.111
5.967	6.021	UDP-NAG	2.660	2.568	3.379	2.786	2.794	2.535	2.870	2.258	2.239	1.777	2.544	2.299	2.760	3.841	2.885	2.778	3.031	2.706	0.104
0.982	1.005	valine	3.874	5.999	5.685	5.506	6.818	5.681	6.766	7.107	6.610	3.861	6.432	4.875	6.051	4.850	5.165	6.534	6.276	5.770	0.219
1.027	1.058	valine	4.087	5.156	5.395	5.599	5.975	5.169	6.295	7.230	7.170	3.485	5.725	4.354	4.966	5.651	4.669	6.547	5.772	5.485	0.227
