# Supplementary material for: Metabolic fingerprint of insulin resistance in human polymorphonuclear leucocytes
Source: PLoS One. 2018 Jul 13;13(7):e0199351. doi: 10.1371/journal.pone.0199351 (PMC6044522; doi:10.1371/journal.pone.0199351)
Supplement: S5 Table — (PDF) [file pone.0199351.s005.pdf]

	Control		Obese		
	mean	SEM	mean	SEM	p
Age	40,1	3,6	43,7	4,8	0,317
BMI, kg/m2	23,9	2,1	40,8	4,7	< 0.0001*
Waist, cm	78,9	3,8	118,5	16,0	0,0002*
Glucose, mg/dL	87,3	3,7	94,1	17,8	0,1674
HDL, mg/dL	79,4	2,2	39,3	16,5	0,0003*
LDL, mg/dL	78,1	8,8	110,9	14,8	0,0107*
TG, mg/dL	115,3	34,7	140,6	17,2	0,7042
Insulin, mg/dL	9,0	3,3	17,8	1,2	0,0331*
HOMA-IR	2,0	0,7	4,1	0,3	0,0131*
