# Supplementary material for: Metabolic fingerprint of insulin resistance in human polymorphonuclear leucocytes
Source: PLoS One. 2018 Jul 13;13(7):e0199351. doi: 10.1371/journal.pone.0199351 (PMC6044522; doi:10.1371/journal.pone.0199351)
Supplement: S6 Table — (PDF) [file pone.0199351.s006.pdf]

	Control (n=10)		Obese (n=10)		
	mean	SEM	mean	SEM	p
3-hydroxyisovalerate	9,27	1,05	5,99	0,67	0,0183*
2-aminoisobutyric acid	5,09	0,60	6,16	0,45	0,0535
acetate	2,18	0,29	2,92	0,30	0,1489
β-alanine	0,45	0,11	0,10	0,10	0,0448*
glycine	11,45	4,67	11,23	0,74	0,0049*
inosine	0,22	0,11	0,05	0,09	0,2751
