# Supplementary figures and images for: Metabolic fingerprint of insulin resistance in human polymorphonuclear leucocytes
Source: PLoS One. 2018 Jul 13;13(7):e0199351. doi: 10.1371/journal.pone.0199351 (PMC6044522; doi:10.1371/journal.pone.0199351)

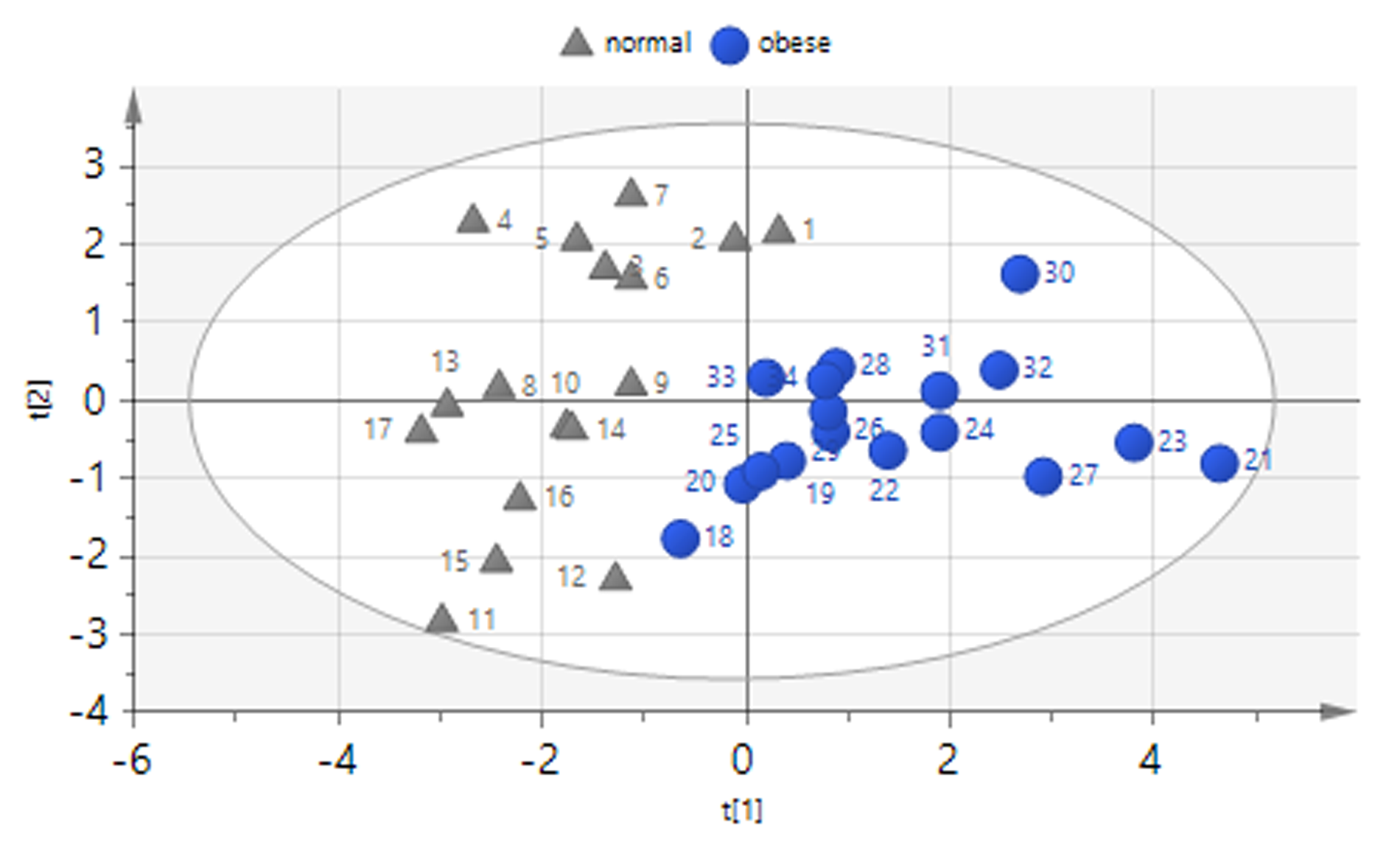

Supplement: S1 Fig — Input variables are age, the BMI, waist, HDL, LDL, TG glucose, insulin and HOMA-IR. R2X = 0.65, Q2 = 0.30. (TIF) [file pone.0199351.s007.tif]

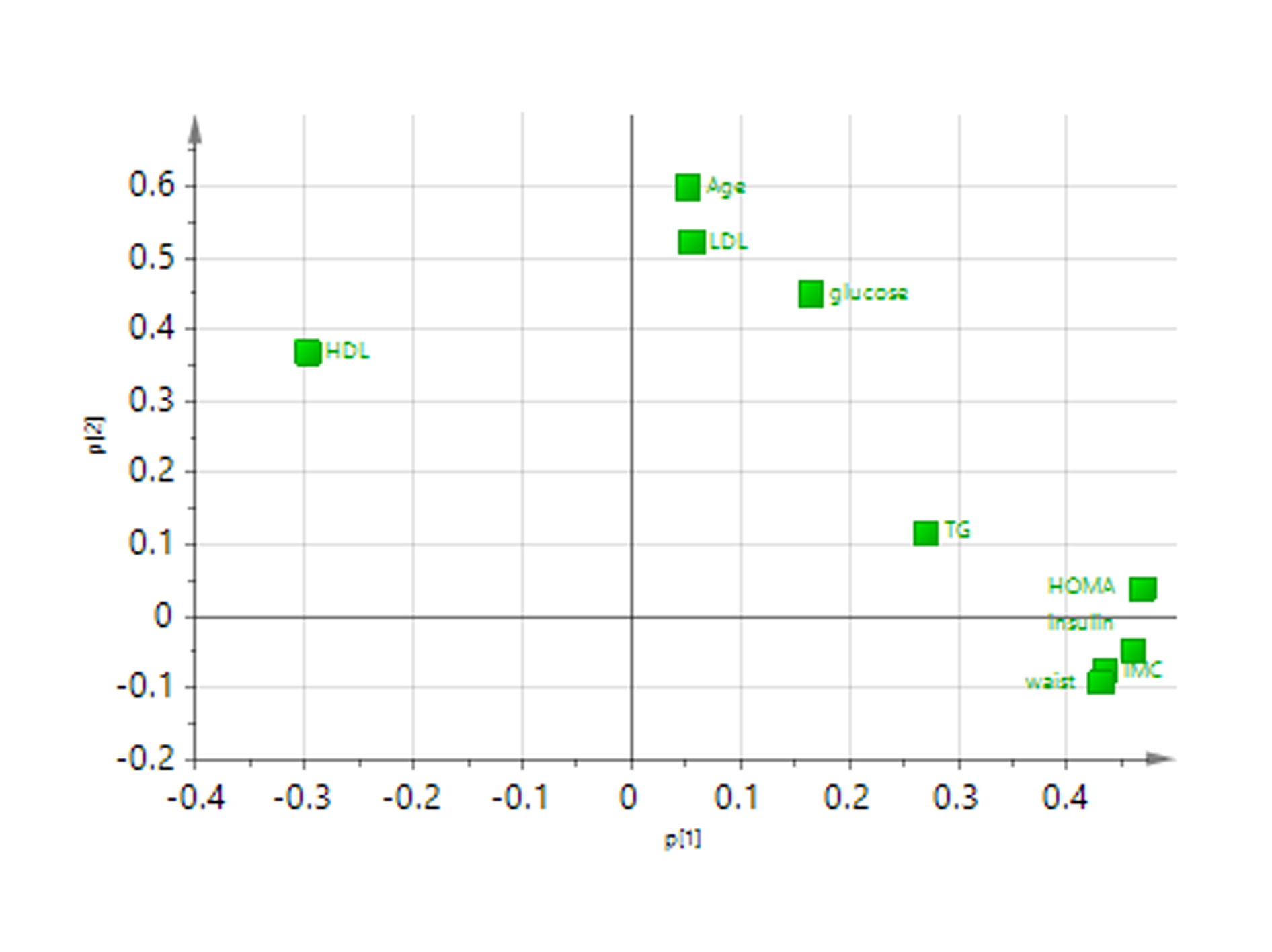

Supplement: S2 Fig — Input variables are age, the BMI, waist, HDL, LDL, TG glucose, insulin and HOMA-IR. (TIF) [file pone.0199351.s008.tif]

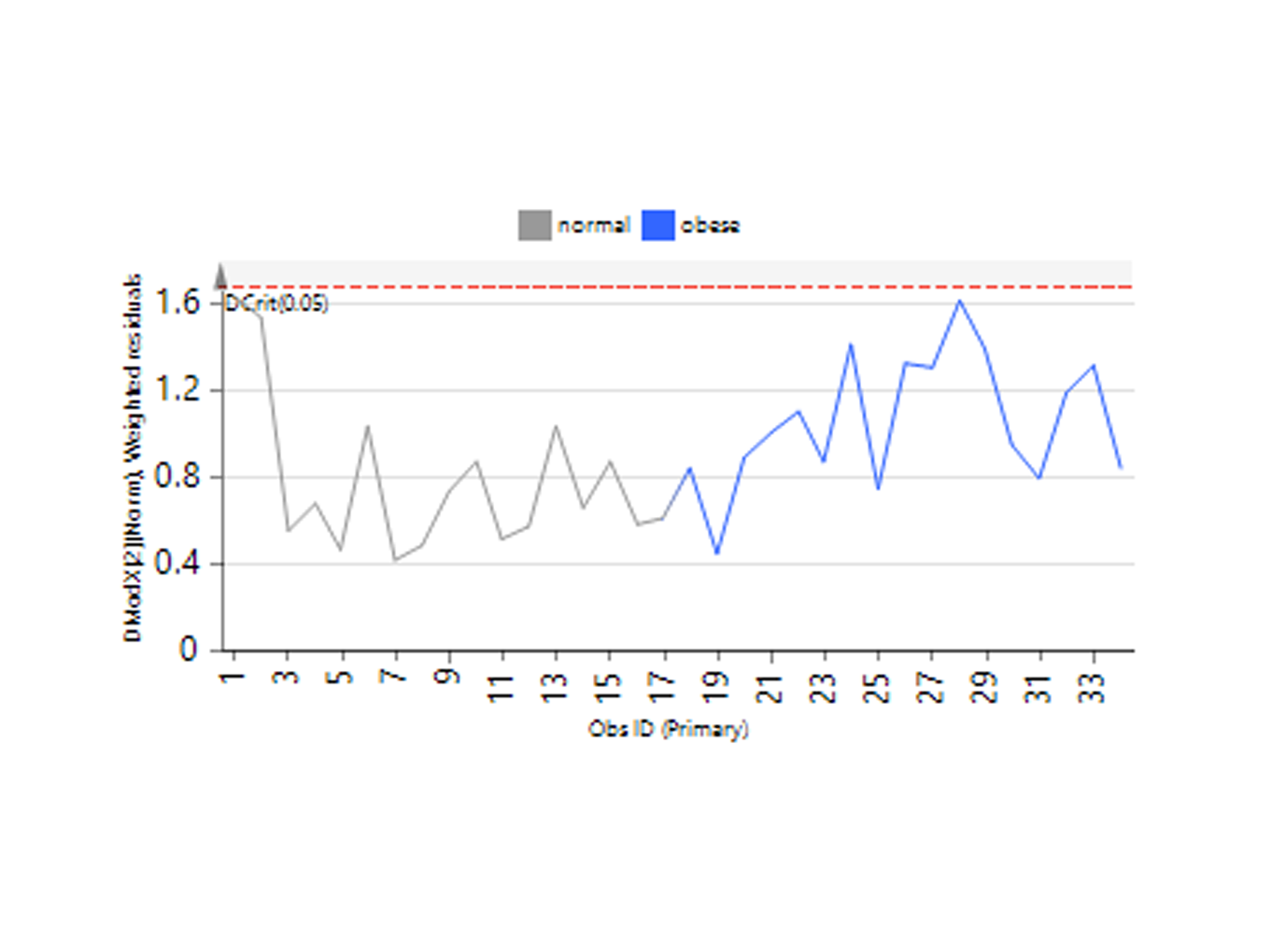

Supplement: S3 Fig — Input variables are age, the BMI, waist, HDL, LDL, TG, glucose, insulin and HOMA-IR. (TIF) [file pone.0199351.s009.tif]

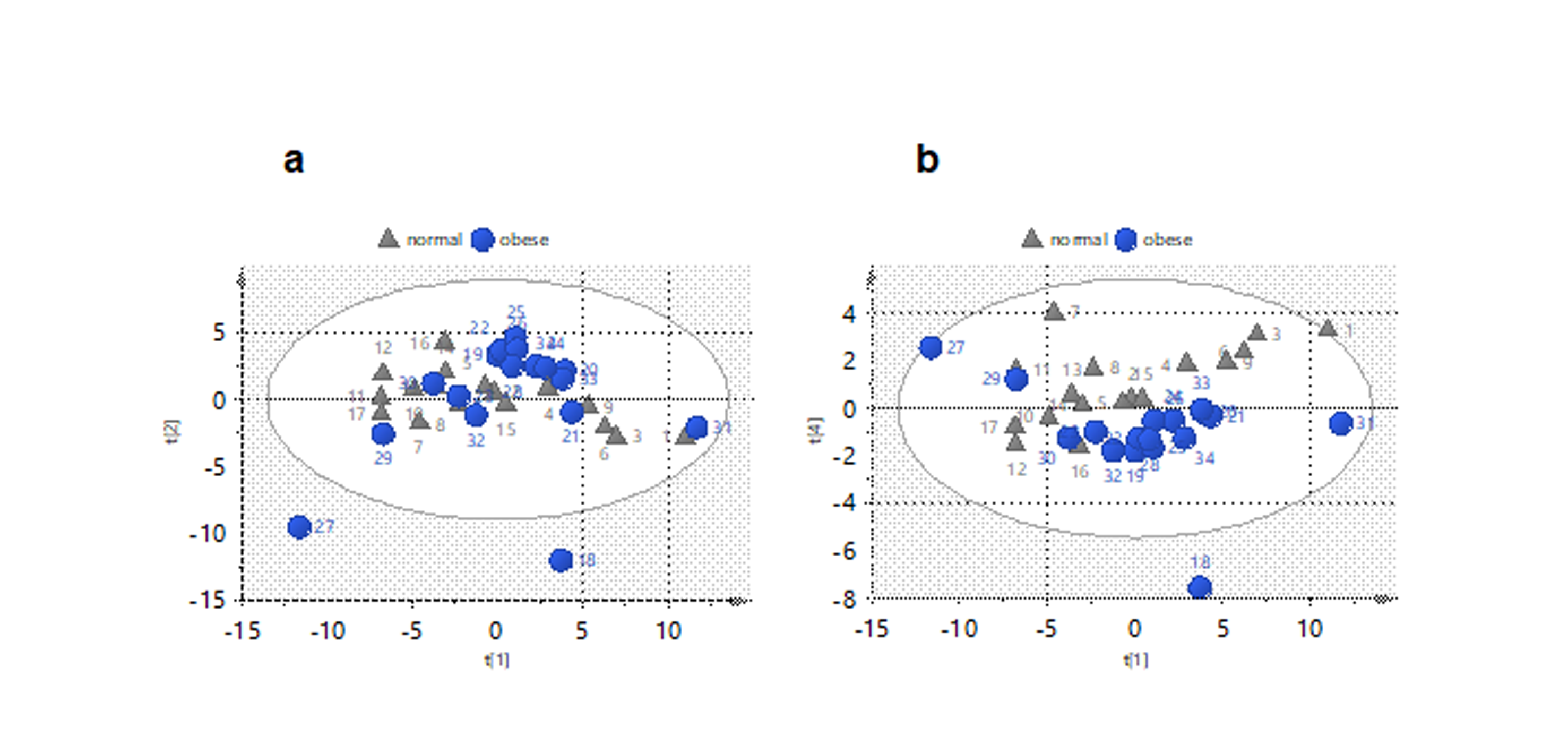

Supplement: S4 Fig — A) Score plot of components 1 and 2. B) Score plot of components 1 and 4. Principal Component 1: R2X = 0.37, Q2 = 0.28. Principal Component 2: R2X = 0.16, Q2 0.07. Principal Component 3: R2X = 0.13, Q2 0.18. Principal Component 4: R2X = 0.06, Q2 0.01. (TIF) [file pone.0199351.s010.tif]

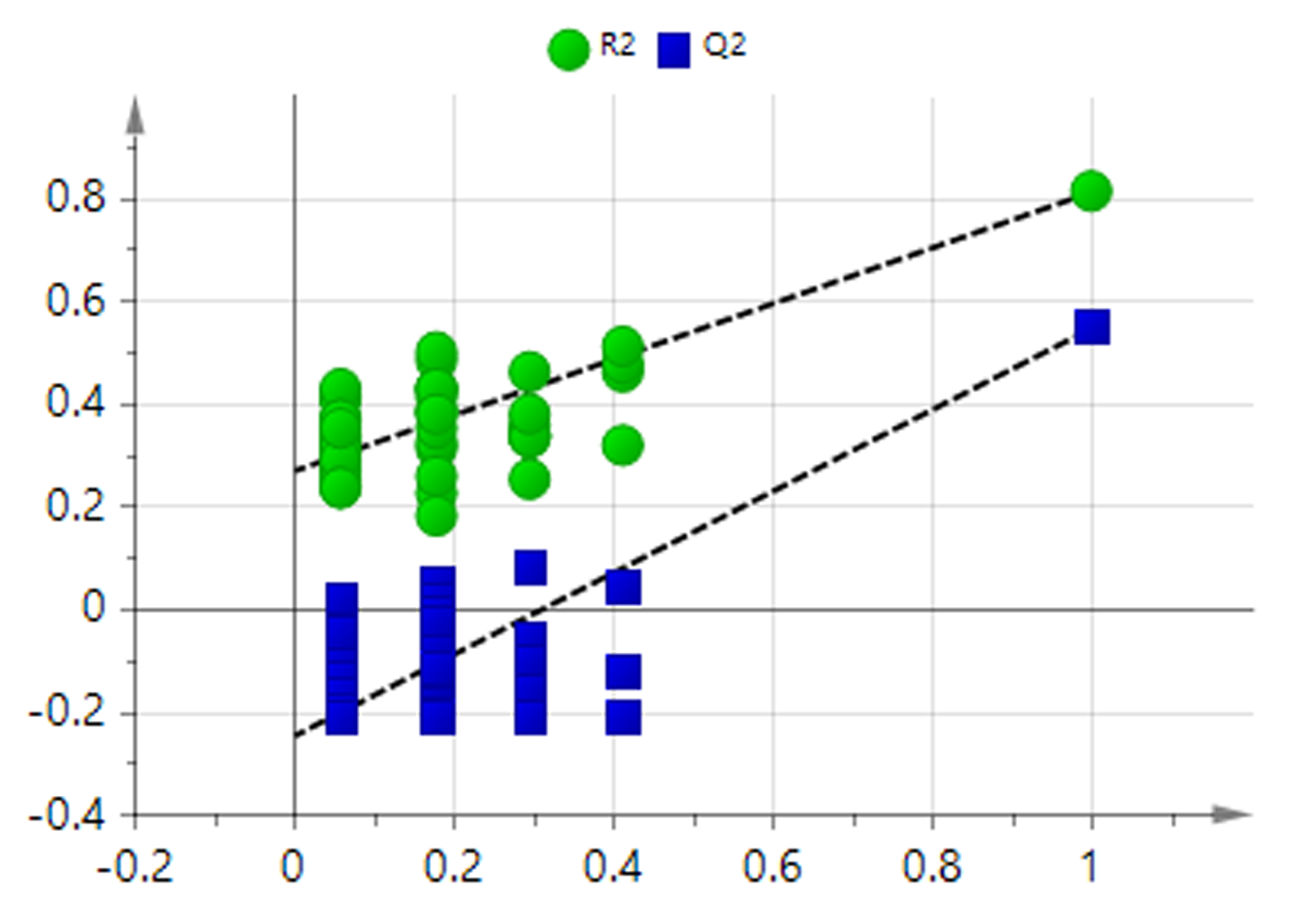

Supplement: S5 Fig — Intercepts: R2 = 0.0, 0.268 Q2 = 0.0, -0.247. PLS-DA model values: R2 = 0.812, Q2 = .0.547. (TIF) [file pone.0199351.s011.tif]

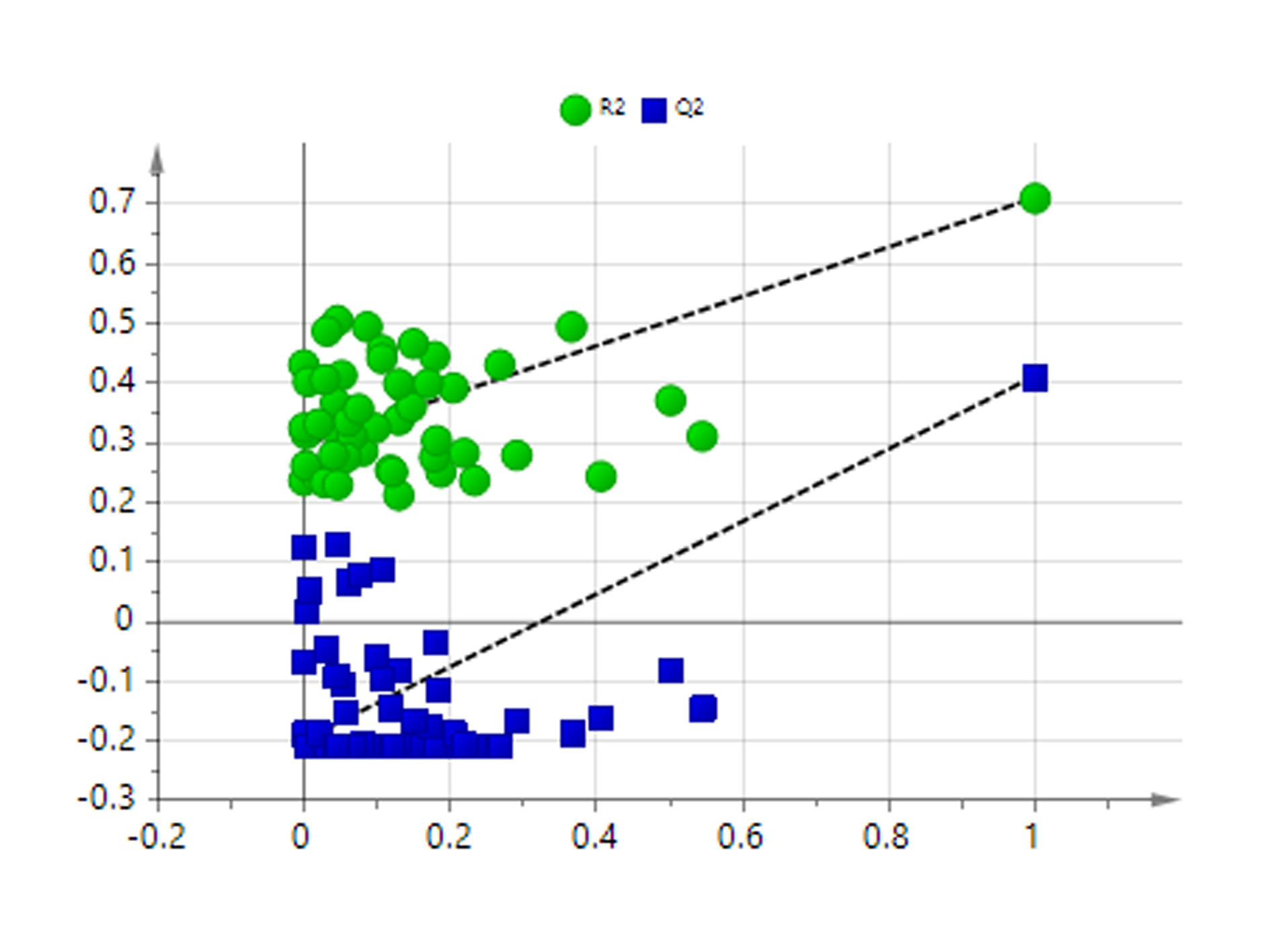

Supplement: S6 Fig — Intercepts: R2 = 0.0, 0.295, Q2 = 0.0, -0.199. Model values: R2 = 0.709, Q2 = 0.410. (TIF) [file pone.0199351.s012.tif]

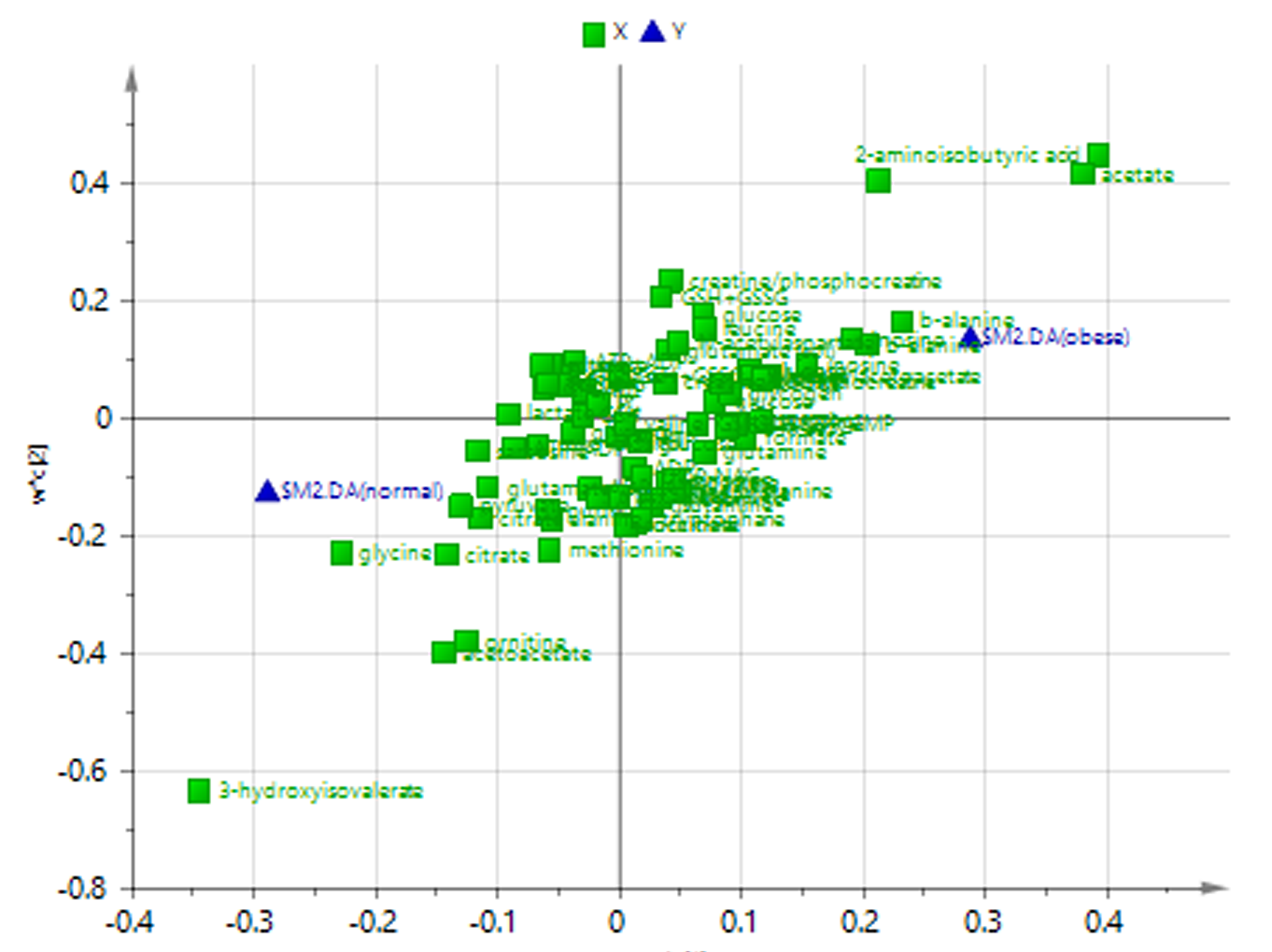

Supplement: S7 Fig — (TIF) [file pone.0199351.s013.tif]

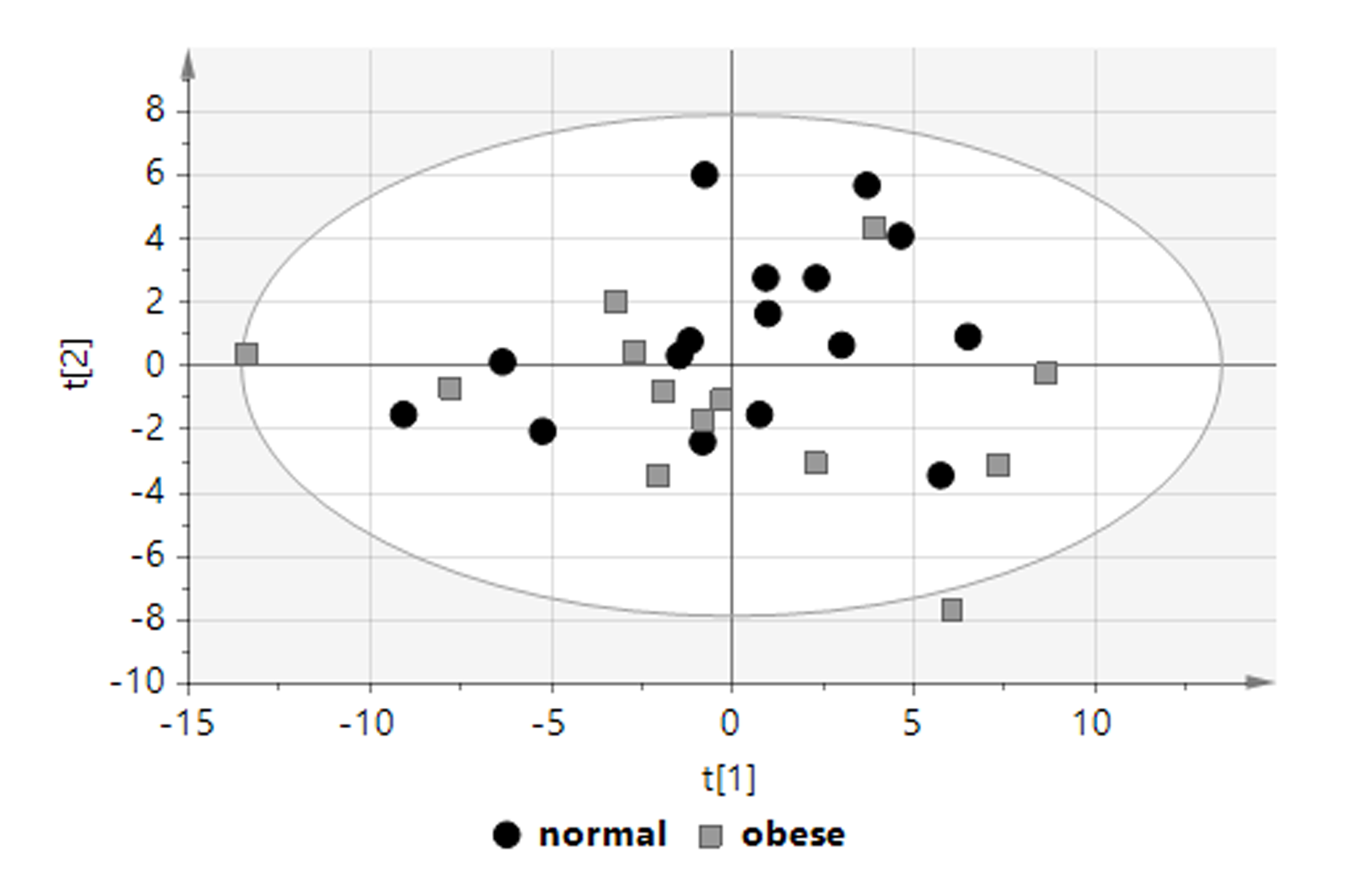

Supplement: S8 Fig — (TIF) [file pone.0199351.s014.tif]
